# Determination of *N*-Carbamylglutamate in Feeds and Animal Products by High Performance Liquid Chromatography Tandem Mass Spectrometry

**DOI:** 10.3390/molecules24173172

**Published:** 2019-08-31

**Authors:** Yonghang Ma, Zhengcheng Zeng, Lingchang Kong, Yuanxin Chen, Pingli He

**Affiliations:** State Key Laboratory of Animal Nutrition, College of Animal Science and Technology, China Agricultural University, Beijing 100193, China

**Keywords:** *N*-carbamylglutamate, feeds, animal products, milk, HPLC-MS/MS

## Abstract

*N*-carbamylglutamate (NCG), a synthetic analogue of *N*-acetylglutamate, is an activator of blood ammonia conversion and endogenous arginine synthesis. Here, we established an accurate quantitative determination of NCG in feeds, animal tissues, and body fluids using the high performance liquid chromatography tandem mass spectrometry (HPLC-MS/MS). The sample pretreatment procedures included extraction with 0.5% of formic acid in water/methanol (80/20, *v*/*v*), and purification using an anionic solid phase extraction cartridge. Satisfactory separation of NCG was achieved in 20 min with the application of an Atlantis T3 column, and a confirmative detection of NCG was ensured by multiple reaction monitoring of positive ions. NCG spiked in feeds, tissues, and body fluids were evaluated in regard to linearity, sensitivity, recovery, and repeatability. Recoveries for different sample matrices were in the range of 88.12% to 110.21% with relative standard deviations (RSDs) less than 8.8%. Limits of quantification were within the range of 0.012 to 0.073 mg kg^−1^ and 0.047 to 0.077 μg mL^−1^ for solid and liquid samples, respectively. This study will provide a solid foundation for the evaluation of availability and metabolic mechanism of NCG in animals.

## 1. Introduction

**Figure 1:**
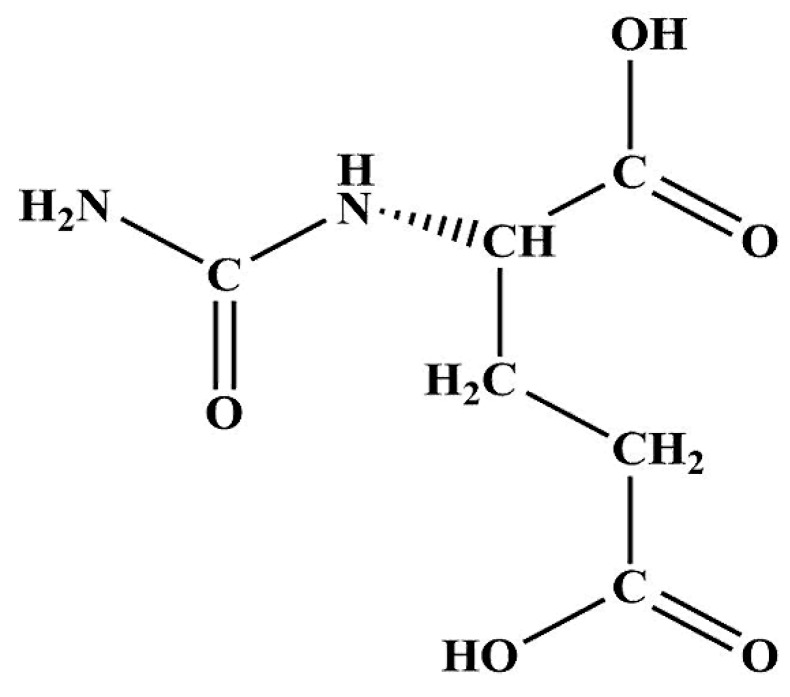
The chemical structure of *N*-carbamylglutamate.

*N*-carbamylglutamate (NCG) is a synthetic analogue of *N*-acetylglutamate (NAG) (Figure 1), which is the allosteric stimulator of carbamyl phosphate synthase-1 (CPS1). CPS1 is a key enzyme functioning in the urea cycle and endogenous arginine synthesis pathway [1]. Like NAG, NCG can activate CPS1 and lead the conversion of blood ammonia into the mitochondrial carbamoyl-phosphate, further stimulating the endogenous synthesis of arginine. Due to its good stability and safety [2,3], NCG was initially used for the clinical treating of hyperammonemia caused by the NAG synthetase deficiency [4,5], propionic aciduria and methylmalonic aciduria [6,7], and maple syrup urine disease [8]. NCG is also proven to be a novel, effective and low-cost substitute feed additive for arginine. In 2014, China’s ministry of agriculture approved NCG as a new feed additive to use in the livestock (new feed additive certificate no. 2014-01). The maximum addition limit of NCG in the compound feed is 800 g t^−1^ of sow, and 880 g t^−1^ of dairy cow, respectively. Compared with the sole supplementation of NAG or arginine, NCG can avoid being catabolized by the deacylase or amino acid metabolism enzymes, and does not cause nutritional antagonism against other amino acids, especially lysine, tryptophan, and histidine [1,9]. Previous studies have indicated that oral administration of NCG 50 mg per kg body weight two times a day, increases piglets’ plasma arginine concentration by 68%, and weight gain by 61% in 10 days [1]. More recently, several results have been reported that gilts with dietary supplementation of 0.05% and 0.1% of NCG had more pigs born alive, and significantly increased the live litter weight [10,11]. A similar effect of NCG on growth promotion and reproductive performance enhancement have also been found in other species of animals including the rat [12], cattle [13], sheep [14], goat [15], and yellow-feather broiler [16]. 

Although the physiological functions of NCG have been well elucidated, and NCG has long been shown to be non-toxic and free of side effects [2], its metabolic process in animals remains unclear. With the promising use of NCG in the livestock, it is necessary to develop an accurate measurement method for NCG in the feed and animal products. Based on the chemical structure of NCG, the infrared spectroscopy (IR), nuclear magnetic resonance (NMR) spectroscopy, and high performance liquid chromatography (HPLC) have been adopted in the analysis of the structure and content of the NCG additive (purity > 97%) [17]. However, due to the complexity of the feed and animal products matrices, few studies have been conducted to deal with the determination of NCG in the feeds [18], and to the best of our knowledge, there is no former report regarding the NCG residue detection in animal tissues or body fluids. 

Since NCG has poor absorption of the UV-Vis spectrum, using the HPLC based on the UV detection will result in low responsivity and sensitivity. Our group tried to determine the NCG using the before- or past-column derivation, but it showed a multiple matrix interference leading to unsatisfactory resolution results, and the procedures were relatively complex. In recent years, the high performance liquid chromatography-tandem mass spectrometry (HPLC-MS/MS), due to its high selectivity and sensitivity, has become the preferred method to analyze the trace small molecular compounds in the complex substrates [19,20]. This study developed a HPLC-MS/MS method to qualitatively and quantitatively determine NCG in the feed, animal tissues, and body fluids with excellent accuracy and sensitivity. The proposed method can achieve a fast separation of NCG within a 20 min gradient elution, and the method validation was achieved by evaluating the linearity, sensitivity, recovery, as well as the accuracy and repeatability for NCG in the feeds and animal products.

## 2. Results and Discussion

### 2.1. Optimization of HPLC-MS/MS Conditions

**Figure 2:**
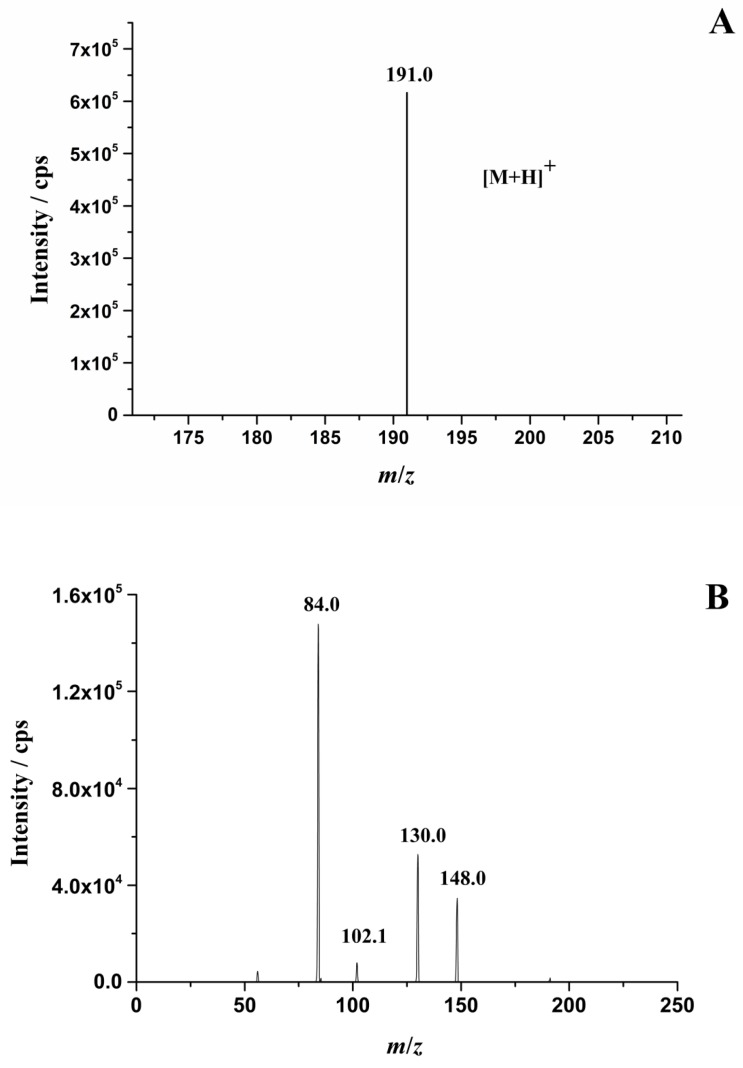

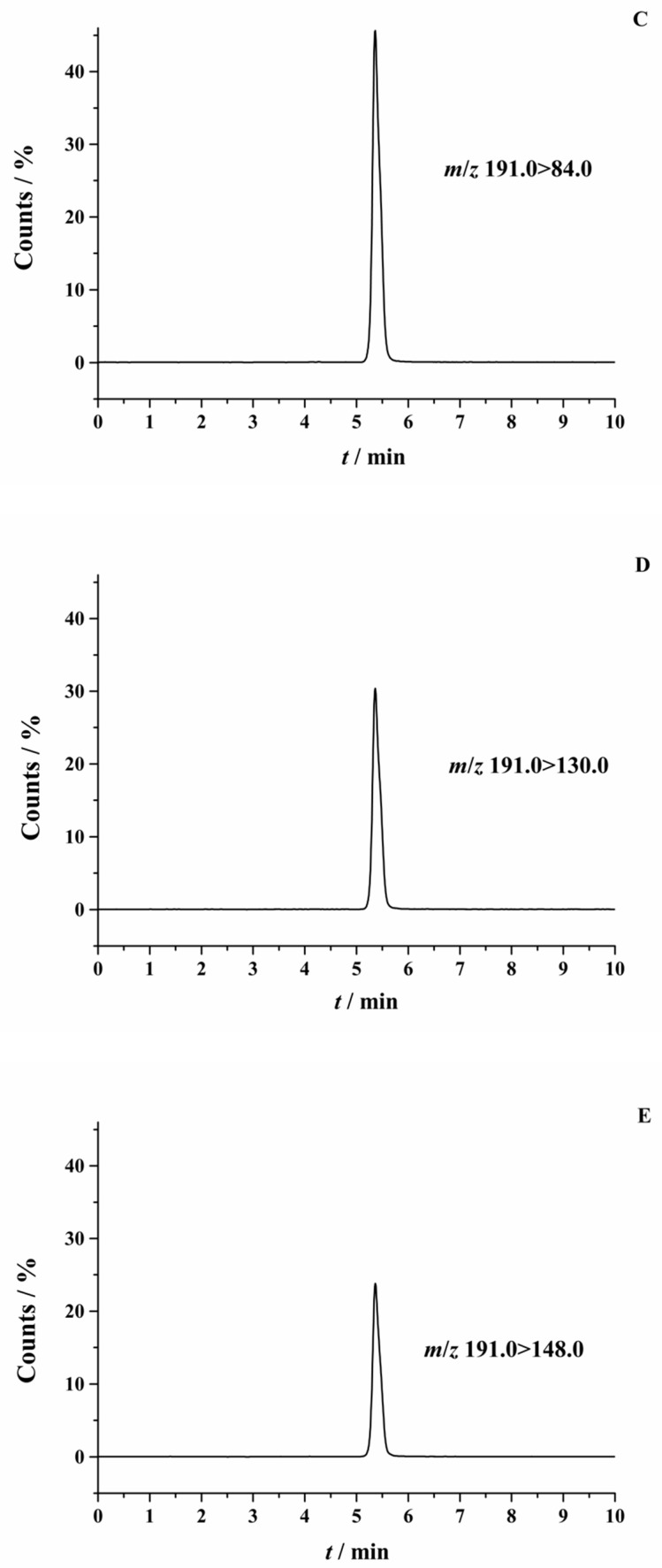
(**A**) Parent ion chromatogram of *N*-carbamylglutamate (NCG); (**B**) the tandem mass spectrometry (MS/MS) spectrum of the NCG precursor ion; (**C**–**E**) typical extracted mutiple reaction monitoring (MRM) ion chromatograms of the NCG standard conducted under optimal conditions.

In order to improve the analytical sensitivity and selectivity, parameters of the mass spectrometry such as the ionization mode, source temperature, capillary voltage, nebulizer pressure, sheath gas flow, collision energy, and fragmentor voltage were optimized using the NCG standard solution. The result showed that the most abundant precursor ion of NCG was the pseudo-molecular ion [M + H]^+^ at *m*/*z* 191.0 (Figure 2A and Figure S1). The MS/MS spectrum of this precursor ion is shown in Figure 2B. The most abundant product ion (*m*/*z* 84.0) was used for quantitation while the two relatively abundant product ions (*m*/*z* 130.0, *m*/*z* 148.0) were selected for qualification (Figure 2C–E). With the aim to increase the signal responses of characteristic product ions and their stability, the parameters (fragmentor, collision energy) in the second mass analyzer were optimized. The optimal fragmentor of the precursor ion (*m*/*z* 191.0) is 50 V. The optimal collision energy of *m*/*z* 84.0, *m*/*z* 130.0, and *m*/*z* 148.0 are 15 V, 10 V, and 5 V, respectively.

NCG is a compound with strong polarity. A Waters Atlantis T_3_ C_18_ column (4.6 × 250 mm, 5 μm) was compared with a Waters BEH C_18_ column (2.1 × 100 mm, 1.7 μm). The T_3_ column showed a better retention for polar compounds with reduced peak tailing thus better peak shapes and improved analytical results than the BEH column. In addition, considering that there are many free amino acids, fatty acids, and other small molecular metabolites in animal samples, the long analytical column with strong polarity is more suitable for the separation of NCG without a precolumnar derivation. Thus, the Atlantis T3 C18 column was chosen as the analysis column. Moreover, the mobile phase of HPLC is a critical factor influencing the analytical results and sensitivity of the detection. Compared with acetonitrile, using methanol as the organic mobile solvent can achieve better chromatographic separation of NCG. Due to the weak acidity of NCG, the addition of a small amount of formic acid could improve the peak shape and improve the sensitivity. Therefore, 0.1% of the formic acid aqueous solution and methanol were selected to be the mobile phases for the binary gradient pump system. Figure 2C showed the typical extracted MRM ion chromatogram of NCG conducted under optimal conditions.

### 2.2. Optimization of Extract Condition

**Figure 3:**
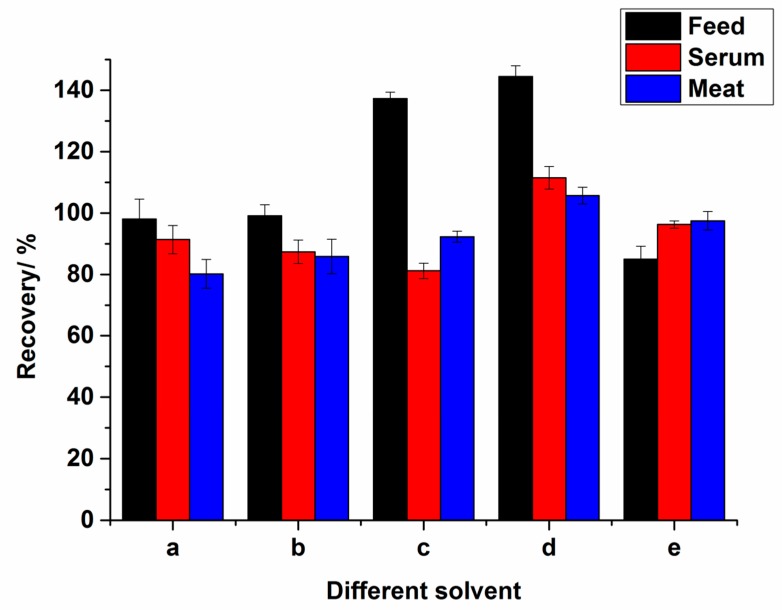
Recoveries of NCG from matrices using different solvents: (a) 0.5% of formic acid solution; (b) 0.5% of formic acid solution/methanol (80:20, *v*/*v*); (c) 0.5% of formic acid solution/methanol (50:50, *v*/*v*); (d) 0.5% of formic acid solution/methanol (20:80, *v*/*v*); (e) methanol.

An effective pretreatment should serve the purpose that the impurity substance is excluded while the target analyte stays as much as possible. As NCG is soluble in both water and methanol, pilot experiments have been conducted to determine the optimal extraction solvent with the finest extraction result. For the feed samples, five extract solvents including water, water/methanol (80:20, *v*/*v*), water/methanol (50:50, *v*/*v*), water/methanol (20:80, *v*/*v*) and methanol were compared. The results show that the recoveries were water > water/methanol (80:20, *v*/*v*) > methanol > water/methanol (50:50, *v*/*v*) > water/methanol (20:80, *v*/*v*). However, using water as the extraction solvent was subjected to a greater matrix effect presumably due to the complex water-soluble additive in the feed. Thus, water/methanol (80:20, *v*/*v*) was selected as the extraction solvent. To better improve the extraction efficiency of NCG in the water/methanol (80/20, *v*/*v*) solvent, different concentration levels of formic acid in the water including 0, 0.1%, 0.5%, and 1% were compared. The results show that 0.5% of formic acid in water/methanol (80/20, *v*/*v*) manifested the best extraction result with a recovery of 99.1%. In addition, the high recovery (>100%) were shown in Figure 3C and D. We speculated that the matrix effect in the C and D extract may cause the enhancement of the NCG signal in MS, which leads to the recovery of NCG over 100%. Therefore, 0.5% of formic acid in water/methanol (80/20, *v*/*v*) was applied as the extraction solvent for NCG in the feeds (Figure 3). For the animal samples, the biggest interference came from the high lipid and protein content in the samples [21]. Similarly, different proportions of methanol and 0.5% of formic acid solution were also tested for the optimization of extraction agents. Moreover, the application of ice-cold methanol combined with the subsequent refrigerated centrifugation step can efficiently precipitate and remove most of the interference proteins present in milk, serum, and tissue samples [20,22]. Thus, ice-cold methanol (100%) was finalized as the optimal extraction solvent for NCG in the animal body fluids and tissues due to its precipitation effect of proteins and relatively good recovery (Figure 3).

### 2.3. Optimization of Purification Condition

**Figure 4:**
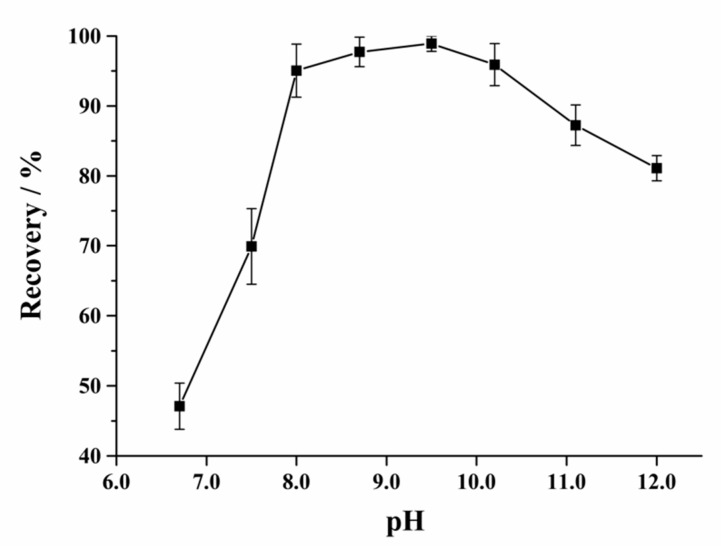
Dependence of the retention abilities of the NCG on the solid-phase extraction (SPE) cartridge with different pH of the loading solvent.

A subsequent purification experiment of the sample extracts was conducted using the solid-phase extraction (SPE) cartridges to further decrease the matrix interference and enhance sensitivity. NCG is a weak acid compound. A strong anion exchange SPE cartridge was used in virtue of its strong adsorption ability for the anionic compound. For the better retentivity of NCG, an appropriate amount of 5% ammonia solution was added to adjust the pH of the extract and make NCG anionic. The results show that the pH of the extract that ranged from 8 to 10 was optimal, the NCG retained above 95% on the SPE (Figure 4). Since the cartridge was a strong anion exchange column, considering the isoelectric point of the NCG (pI 3.02), a lower pH (<8) will not be able to sufficiently make the NCG anionic, resulting in lower retentivity. On the other hand, if the pH is too high (>10), other interfering compounds may be turned anionic and compete with NCG for the limited retention capacity of the cartridge, this will also cause lower retentivity of NCG in the column. In addition, the NCG content in the feeds were very high, a direct application of the sample extracts may cause oversaturation and lead to unsatisfactory recoveries of NCG due to limited adsorption capacity of the cartridges. Our study has indicated that a reduction of the NCG concentration in the sample extracts could significantly increase the NCG recoveries. To avoid oversaturation and improve the peak shape, feed sample extracts were diluted 10 times with water before loaded onto the cartridges. On the contrary, if the content of the NCG in the sample is too low, such as the biological sample, the loading volume should be increased before passing through the column.

### 2.4. Method Validation

#### 2.4.1. Stability and Matrix Effect

For the stability test, the NCG stock solution was diluted into 0.005, 0.1, 2 μg mL^−1^ to have six aliquots with concentrations around the linearity range. These aliquots were stored at −4 °C and 20 °C, respectively. Normally, the criterion for the stability is that degradation is equal to or less than 5%. The results show that the solution could be stored stably for at least three months and one week at the −4 °C refrigerator and 20 °C, respectively. 

Matrix effects come from various chemical and physical processes during ionization of the analytes in the ESI mode of mass spectrometry, which causes signal suppression or enhancement. The matrix effect was evaluated by comparing three NCG concentrations (0.005, 0.1, 2 μg mL^−1^) constructed in the solvent and fortified sample extract. The matrix effects were calculated by comparing the peak area of NCG in the solvent with the peak area of NCG in the fortified sample extract. The results show that the matrix effects ranged from 1.08 to 0.54 for the feed and biological samples. Especially for the biological samples, the matrix inhibition effects were obvious when a high concentration NCG was added. Therefore, the quantification of NCG must be performed using the matrix-matched calibrators.

#### 2.4.2. Linearity, Precision, Accuracy, LOQ and LOD

A series of experiments were carried out with the aim to validate the linearity, sensitivity, precision, and accuracy of the established method. The NCG stock solution was diluted into 0.0016, 0.08, 0.2, 0.5, 1, 2 μg mL^−1^ working solutions. Excellent linearity was obtained at the NCG concentrations ranging from 0.0016–2 μg mL^−1^ with a correlation coefficient of (R^2^) 0.999. To evaluate the sensitivity of this method, the limits of detection (LOD) and the limits of quantitation (LOQ) were tested. The LOD, defined as the analyte concentration at three times the signal to noise ratio (S/N), was determined for NCG in the feed, milk, serum, meat, liver, and kidney samples. The results indicated that the LOD values were within the range of 0.0035 to 0.022 mg kg^−1^ and 0.014 to 0.023 μg mL^−1^ for the solid and liquid samples, respectively (Table 1 and Table 2). The LOQ, defined as the analyte concentration at 10 times the S/N, were in the range of 0.012 to 0.073 mg kg^−1^ and 0.047 to 0.077 μg mL^−1^ for the solid and liquid samples, respectively (Table 1 and Table 2). Here, the compound feed, concentrated feed, and premixed feed are all called the feed. Since the three matrices are similar, the LOD and LOQ values for the three feeds were just slightly different. For convenient use, we have integrated the three similar results into one LOD and one LOQ.

**Figure 5:**
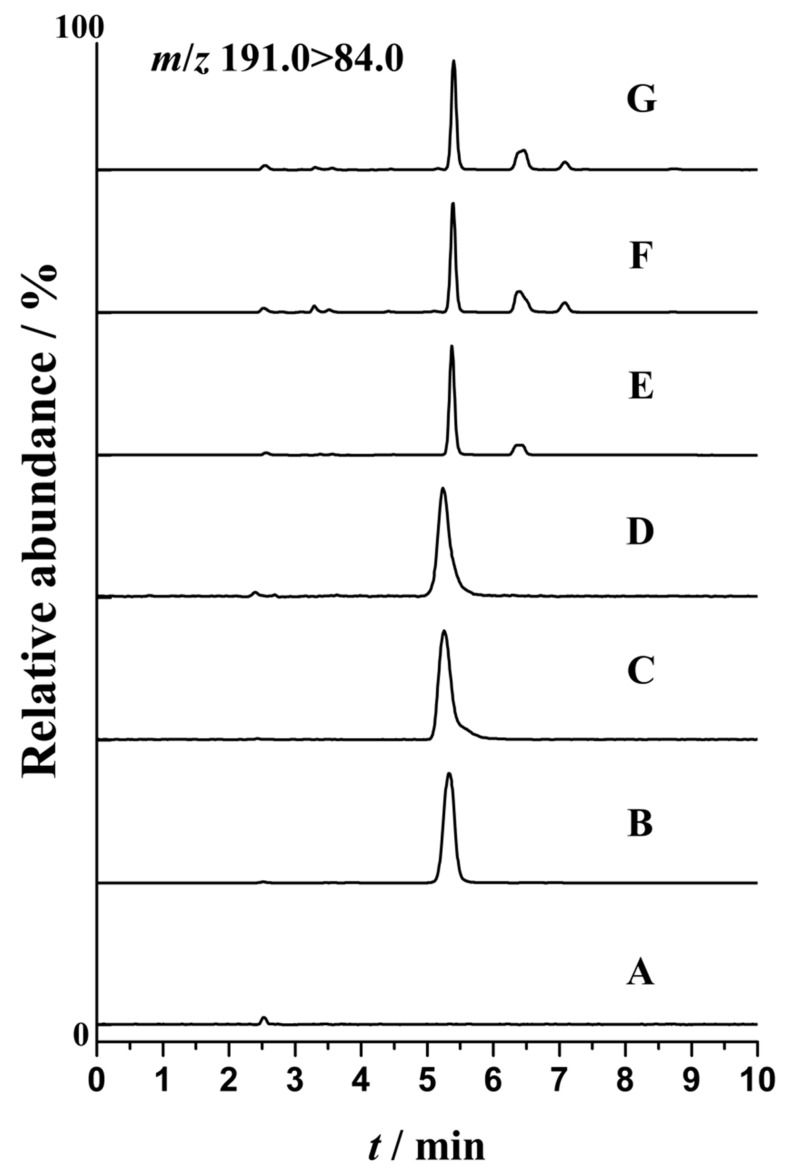
Quantitative product ion chromatograms of the NCG in different matrices: (**A**) Blank feed sample; (**B**) compound feed; (**C**) milk; (**D**) serum; (**E**) meat; (**F**) liver; (**G**) kidney.

The method precision and accuracy were evaluated by the recoveries of NCG in the spiked samples. Recoveries were determined for the feeds, milk, serum, meat, procine liver, and kidney, three replicates were used for each sample at different concentrations. The MRM ion chromatograms for a blank (free of NCG) feed sample and the compound feed sample spiked with NCG were demonstrated in Figure 5A,B and Figures S2–S19, respectively. The peak intensity ratio of the three product ions of NCG can be used to confirm the presence of NCG when compared with the peak intensity ratio obtained from the standard sample. The most abundant product ion of the mass spectrum was selected for the quantitative determination and evaluation of the recoveries for NCG. Calibration curves developed using an external standard were used to quantify NCG in the spiked samples. The spiked levels, spiked recoveries, and coefficients of variation were evaluated. According to the recommended dose of NCG in the feeds, NCG was spiked in the compound feed at 0.01%, 0.05%, and 0.1%, the premix feed at 0.05%, 0.2%, and 1%, and the concentrate feed at 0.5%, 2%, and 10%. Three replicates were analyzed for each concentration. A good consistency was found between the actual spiked amount of NCG and the estimated concentrations in three different feed matrices (Table 1). The recoveries were within the range of 91.90% to 107.0% with the coefficients of variation ranging from 1.3% to 8.8%.

Similarly, the above established method was also applied to the detection of the NCG spiked animal products. The MRM ion chromatograms for the blank and spiked animal products samples were shown in Figure 5C–G and Figures S20–S49, respectively. NCG was spiked in meat, kidney, and liver at 0.05, and 0.1 mg kg ^−1^. For each sample concentration, three replicates were analyzed to evaluate the spiked levels, spike recoveries, and coefficients of variation. As presented in Table 2, reliable results were found for most test samples. The recoveries for NCG ranged from 89.14% to 101.56% and the coefficients of variation were less than 7.6%. The NCG were spiked in milk and serum at 0.1, 1, and 10 μg mL^−1^. The recoveries ranged from 88.12% to 110.21% with the coefficients of variation ranging from 1.8% to 5.1% (Table 2).

Both the intra-assay and inter-assay reproducibility of this method were evaluated. For instance, five repeats at an intermediate concentration (0.05%) were used to determine the recoveries of NCG spiked in the compound feed. According to the analytical results, the recoveries of NCG were within the range of 98.89% to 102.76% and the coefficients of variation were less than 2.9% and 3.8% for the intra-assay (within a day) and inter-assay (over a period of five consecutive days) measurements, respectively. For the animal samples, this method also showed satisfactory reproducibility. For 1 μg mL^−1^ of NCG spiked in the serum, the recoveries of NCG were within the range of 93.25% to 104.68% and the coefficients of variation were less than 4.7% and 5.4% for the intra-assay and inter-assay measurements, respectively. For 0.05 mg kg^−1^ of NCG spiked in the liver, the recoveries of NCG were within the range of 91.23% to 106.71% and the coefficients of variation were less than 3.9% and 6.7% for the intra-assay and inter-assay measurements, respectively.

### 2.5. The Analysis of Authentic Samples

The method established in this study was applied to determine the NCG content in the authentic feed samples added with NCG additive. The results showed that NCG was detected in the compound feed, concentrated feed, and premix feed samples with the average content of 0.098%, 0.95%, and 9.53%, respectively. The variation between the two parallel samples was less than 10%, while NCG was not detected in the ordinary swine feed samples (<0.022 mg kg^−1^). According to the actual addition amount, the accuracy for the determination of the compound, concentrate, and premix feed was 98.0%, 95.0%, and 95.3%, respectively. The proposed method was also adopted for the determination of NCG in the authentic serum samples from pigs and milk sample from cows (each concentration has six replicates). The results showed that the concentration of NCG in the serum samples increased significantly with the increase of NCG in the diets (Table 3). Except for the control group, the NCG levels in the serum of four diets ranged from 0.11 to 1.31 μg mL^−1^. Moreover, the multiple relationship between the serum NCG and dietary NCG was basically consistent. The NCG concentration in milk was about 0.0647 μg mL^−1^ when the cow was fed 20 g of NCG per day. Therefore, the developed method proved both feasible and reliable for the quantitative determination of NCG in the routine analysis.

## 3. Materials and Methods

### 3.1. Materials and Reagents

The NCG standard (purity >99.5%) was purchased from Sigma-Aldrich (St. Louis, MO, USA). The methanol and formic acid used were of a HPLC grade and were obtained from Fisher Scientific International (Hampton, NH, USA) and Dikma Technology (Richmond Hill, ON, Canada), respectively. Other chemicals used were all of the analytical grade. A Milli-Q system (Millipore Corporation, Bedford, MA, USA) was applied to provide ultrapure water for the preparation of all aqueous solutions.

The NCG stock solution (1 mg mL^−1^) was prepared by dissolving the NCG standard in ultrapure water, and preserved at 4 °C. The preparation of the NCG working solutions was done daily by diluting the stock solution to appropriate concentrations with ultrapure water. The extraction solvent, 0.5% of formic acid in water/methanol (80/20, *v*/*v*), was prepared by mixing 4 mL of formic acid with 796 mL of Milli-Q water, and then mixing with 200 mL of methanol.

### 3.2. Instruments and Apparatus

The HPLC- MS/MS was performed on an Agilent 1200 UHPLC system coupled with an Agilent 6460 Triple Quadrupole Mass Spectrometer (Agilent Technologies, Fermont, CA, USA). An Ultrasonic Cleaner (Kunshan, China) was used to promote the sample dissolution and extraction. A low temperature high speed centrifuge (Eppendorf, Hamburg, German) was used to centrifuge the samples.

### 3.3. Sample Preparation

Feed samples free of NCG were provided by the Ministry of Agricultural Feed Industry Centre (Beijing, China). The liver and kidney as blanks were from a porcine, and milk was from a cow. They were all purchased from local supermarkets. The serum of the pig as blanks were collected from the animal research station of China Agricultural University (Hebei, China). Prior to the extraction, the feed samples were crushed into about 60 mesh by a small mill. The meat, liver, and kidney tissues were homogenized in a homogenizer for five min. The milk and serum were pre-treated by centrifuging at 14,000 rpm for 10 min at 4 °C, then the supernatant was collected.

### 3.4. Sample Extraction and Purification

Extraction of the feed samples was carried out by adding 20 mL of the extraction solvent (0.5% of formic acid in water/methanol, 80/20, *v*/*v*) into the feeds (2 g for the compound feed, 0.2 g for the concentrated feed and premix). Usually, the recommended amounts of NCG in the pig compound feed, premixed feed, and concentrated feed were about 0.05%, 1% and 10%, respectively. Since the concentration of NCG in the premix or concentrated feed was too high, in order to avoid too much dilution before the HPLC-MS/MS analysis, the sample amount in the premix or concentrated feed was relatively reduced. After the vortex mixing and a 30 min ultrasonic bath, 10 min of centrifugation at 14,000 rpm at 4 °C was completed and the supernatant was collected. For the fluid samples, 2 mL of pre-centrifuged milk or serum was pipetted into a Corning^®^ 15 mL centrifuge tube, and 8 mL of an ice-cold extraction solvent was added. After the vortex mixing, the samples were centrifuged at 14,000 rpm for 10 min at 4 °C and the supernatant collection was done. For the tissue analyses, 2 g of tissues samples (meat, kidney, or liver) were extracted by adding 20 mL of an ice-cold extraction solvent. After a thorough vortex mixing, the samples were placed on ice for 30 min and centrifuged for 10 min at 14,000 rpm at 4 °C, then the supernatant collection was done.

Before further purification, the above supernatant was diluted 10 times using water and adjusted by adding an appropriate amount of 5% of ammonia solution to pH 8–10. The strong anion exchange cartridge (Agilent SampliQ SAX, 200 mg, 3 mL, Agilent Technologies, Fermont, CA, USA) was firstly preconditioned with 3 mL of methanol following by 3 mL of water. Then, 3 mL of the diluted supernatant was loaded and slowly passed through the cartridge with a flow rate of approximately 1 mL min^−1^. After rinsing with 3 mL 5% of the ammonia solution and 3 mL of methanol, elution was carried out with 3 mL 2% of formic acid in methanol. The eluate was then collected and gently evaporated under a nitrogen stream in a 50 °C water bath. The residue was reconstituted with 1 mL 0.1% of an aqueous formic acid solution and filtered by a 0.1 µm Syringe Filter (Tianjin Fuji Science and Technology, China). 10 µL of the sample was injected by the autosampler for the HPLC-MS/MS analysis.

### 3.5. HPLC-MS/MS Conditions

A Waters Atlantis T3 column (4.6 × 250 mm, 5 μm) was applied in the chromatographic separation of NCG. The column temperature was maintained at 35 °C. The mobile phases consisted of solution A (0.1% of an aqueous formic acid solution) and B (methanol), and were delivered at a flow rate of 0.8 mL min^−1^. The total run time was set at 20 min. A gradient elution program was developed as follows: 90% of solution A (initial), with 90–15% of solution A (from 6 to 6.1 min), 15–90% of solution A (from 12 to 12.1 min). Before the next injection, a 7.9 min equilibration was necessary. A calibration curve calculated from the NCG standard solution was used to quantify the NCG content in the spiked samples.

The electron spray ionization source was operated under an optimized condition: Capillary voltage 3500 V, nebulizing gas temperature 350 °C, nebulizing gas flow 5 L min^−1^, sheath gas temperature 350 °C, and sheath gas flow 7 L min^−1^. Positive ions were monitored. The multiple reaction monitor (MRM) mode was applied for the quantitation analysis. Data were collected and processed on the Agilent MassHunter Workstation software (Version B.04.00).

### 3.6. The Method Validation

The method validation was performed with the feeds, serum, meat, liver, and kidney samples. Extracts of the NCG-free samples were used for the preparation of matrix-matched calibration standards. The artificially prepared samples were spiked with the NCG standard solution of 0.01–10% in the feeds, 0.1–10 μg mL^−1^ in fluids, or 0.05–0.1mg kg^−1^ in tissues. The validity of the method including the stability, matrix effect, linearity, sensitivity, as well as the precision and accuracy of the method were evaluated in three replicates. 

### 3.7. The Analysis of Authentic Samples

One hundred kg of the commercially prepared compound feed, concentrated feed and premix were added with the NCG feed additive product (NCG content ≥ 97%) obtained from Ya Tai XingMu company (Beijing, China) to the concentrations of 0.1% for the compound feed, 1% for the concentrated feed and 10% for the premix, respectively.

The animals breeding experiments were carried out at the animal research station of China Agricultural University (Hebei, China). All piglets used in this study were housed and handled according to the established guidelines of China Agricultural University, which were approved by the China Agricultural University Animal Care and Use Committee. Thirty weaned piglets (each 30–40 kg in weight) were randomly assigned to one of the five groups (n = 6 piglets per group). Five pig diets were prepared with NCG added 0%, 0.025%, 0.05%, 0.1%, and 0.2%, respectively. After 26 fed days, blood samples from the precaval veins were collected after fasting 12 h and centrifuged (3000 rpm) for 10 min. Then, the supernatant was transferred into new tubes and stored at −20 °C until use. Milk samples were collected from the cow before and after feeding 20 g of NCG per day.

Thirty weaned piglets (each 30–40 kg in weight) were randomly assigned to one of the five groups (n = 6 piglets per group).

## 4. Conclusions

A fast, sensitive and reliable HPLC-MS/MS method was successfully developed and optimized for the qualitative and quantitative determination of NCG in the feeds, animal tissues, and body fluids. Based on the high selectivity and satisfactory separation result of the HPLC-MS/MS technique, this method can achieve confirmative identification and accurate determination of NCG in the complex matrices including feeds and animal products. Furthermore, the applicability of the established method has been validated using the real feed, serum and milk samples.

## Figures and Tables

**Table 1:** Percentage recoveries and relative standard deviations (RSDs) of NCG in different feed matrices (n = 3).

Matrices	Added Concentration (%)	Recoveries (%)	LOD (mg kg^−1^)	LOQ (mg kg^−1^)
Compound feed	0.01	96.85 (1.3) ^a^	0.022	0.073
	0.05	98.89 (2.9)		
	0.1	99.24 (5.0)		
Concentrated feed	0.05	107.0 (3.5)		
	0.2	106.1 (7.1)		
	1.0	97.60 (8.8)		
Premix	0.5	94.27 (6.8)		
	2.0	91.90 (3.9)		
	10	100.2 (3.6)		

^a^ Figures in bracket represented the relative standard deviation (%).

**Table 2:** Percentage recoveries and relative standard deviations (RSDs) of NCG in different animal samples (n = 3).

Matrices	Added Concentration	Recoveries (%)	LOD (μg mL^−1^)	LOQ (μg mL^−1^)
Serum (μg mL^−1^)	0.1	94.25(3.8) ^a^	0.023	0.077
	1.0	110.2 (2.3)		
	10	96.34 (5.1)		
Milk (μg mL^−1^)	0.1	88.12 (1.8)	0.014	0.047
	1.0	102.5 (3.2)		
	10	89.58 (4.7)		
Meat (mg kg^−1^)	0.05	93.84 (7.6)	0.0038	0.013
	0.1	101.6 (3.4)		
Kidney (mg kg^−1^)	0.05	89.14 (3.1)	0.0069	0.023
	0.1	93.65 (5.6)		
Liver (mg kg^−1^)	0.05	94.54 (7.4)	0.0035	0.012
	0.1	97.36 (2.9)		

^a^ Figures in bracket represented the relative standard deviation (%).

**Table 3:** The results of NCG in the authentic samples (n = 6).

Sample	Diets Added (%)	NCG Concentration (μg mL^−1^)	Intra-Day Precision CV (%)	Inter-Day Precision CV (%)
Serum ^b^	0	ND ^a^	-	-
	0.025	0.112	6.8	8.7
	0.05	0.253	2.4	5.6
	0.1	0.561	7.8	11
	0.2	1.31	12	5.1
Milk	0	ND	-	-
	20 g/day	0.0647	11	7.4

^a^ Not detected, ^b^ Serum from piglets.

## Supplementary Materials

The following are available online at https://www.mdpi.com/1420-3049/24/17/3172/s1.
