# Supplementary material for: Determination of N-Carbamylglutamate in Feeds and Animal Products by High Performance Liquid Chromatography Tandem Mass Spectrometry
Source: Molecules. 2019 Aug 31;24(17):3172. doi: 10.3390/molecules24173172 (PMC6749331; doi:10.3390/molecules24173172)
Supplement: Supplementary file 1 [file molecules-24-03172-s001.pdf]

Article

Determination of N-carbamylglutamate in feeds and animal products by high performance liquid chromatography tandem mass spectrometry

Yonghang Ma, Zhengcheng Zeng, Lingchang Kong, Yuanxin Chen, and Pingli He*

State Key Laboratory of Animal Nutrition, College of Animal Science and Technology, China Agricultural University, Beijing, 100193, China; wyzs1602@cau.edu.cn (Y.M.); 996845493@cau.edu.cn (Z.Z.); 1718217239@qq.com; (L.K.); 2017304010111@cau.edu.cn (Y.C.)

*Correspondence: hepingli@cau.edu.cn; Tel.: +86-10-62733688

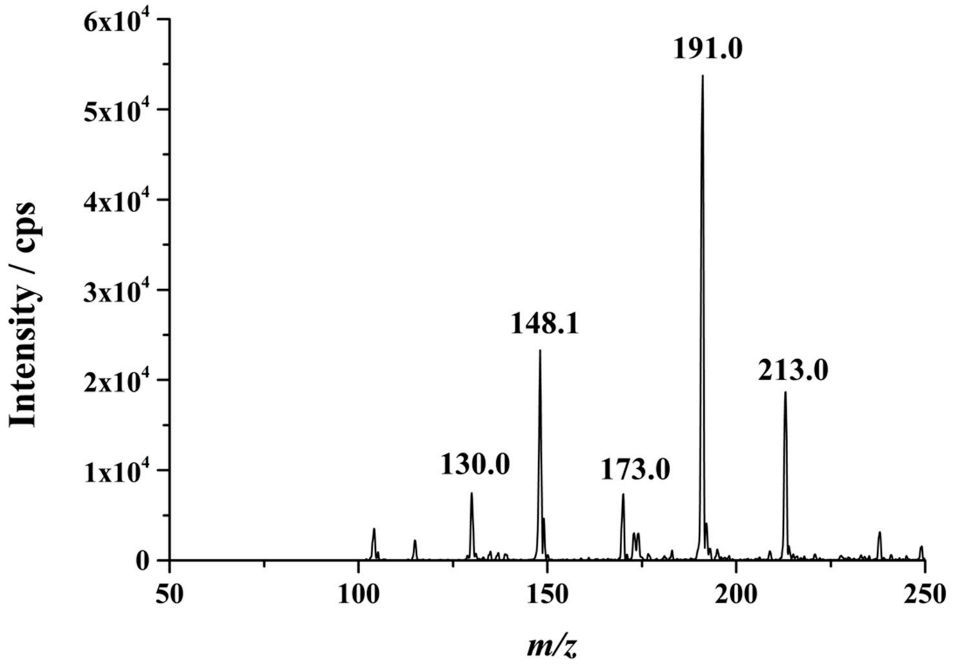

Figure S1. The full scan MS spectrum of NCG standard.

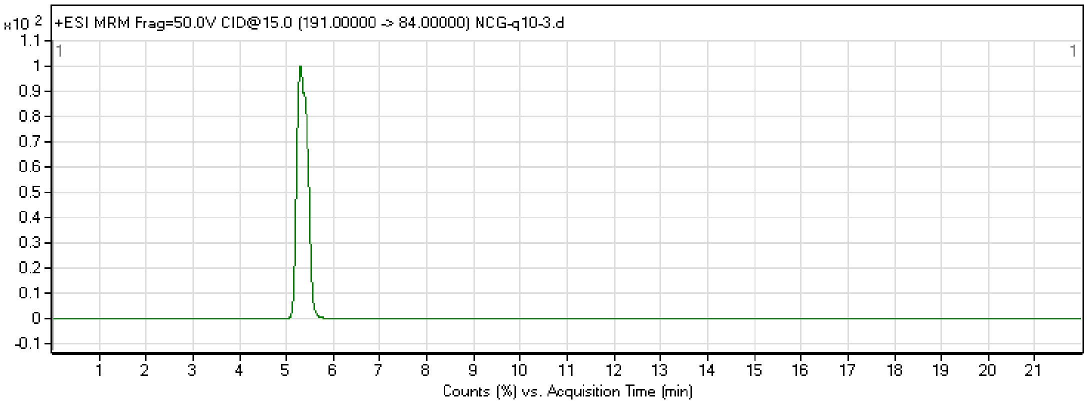

Figure S2. Product ion *m/z* 191.0>84.0 chromatogram of NCG in spiked compound feed.

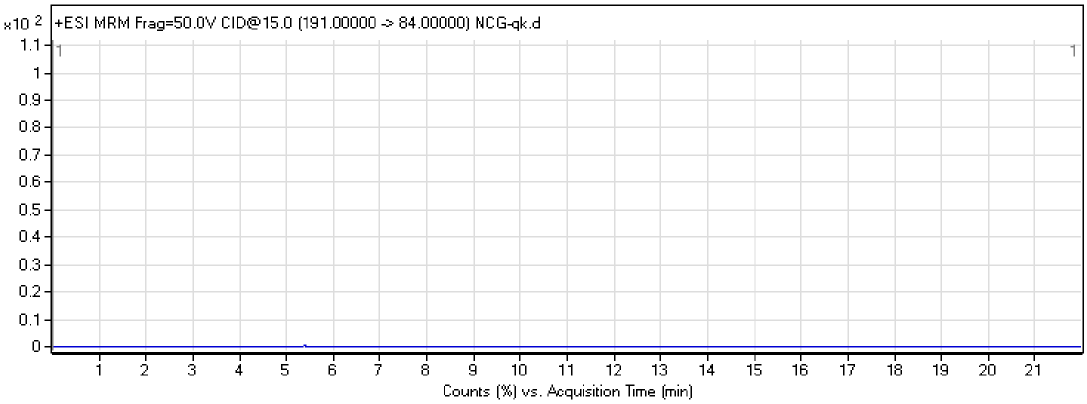

Figure S3. Product ion *m/z* 191.0>84.0 chromatogram of NCG in blank compound feed sample.

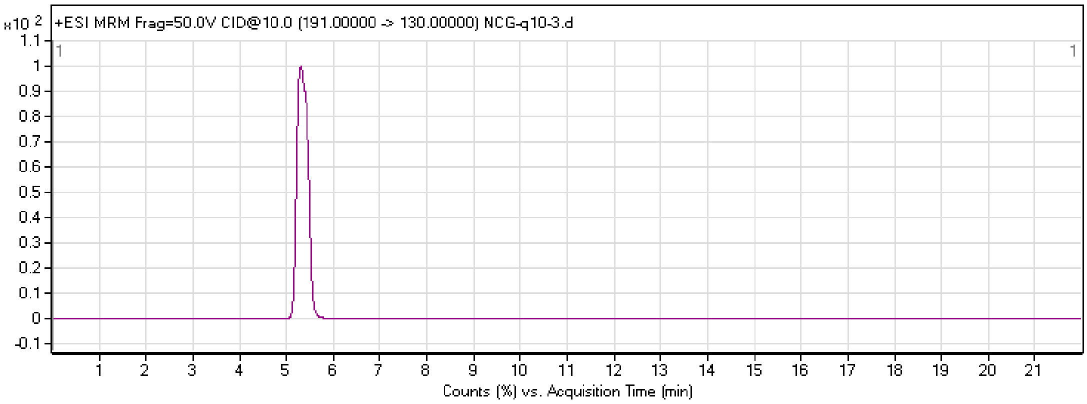

Figure S4. Product ion *m/z* 191.0>130.0 chromatogram of NCG in spiked compound feed.

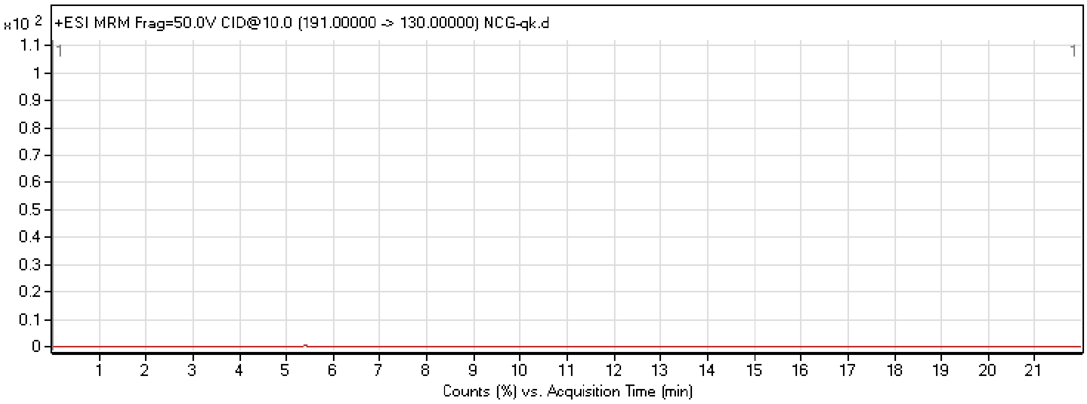

Figure S5. Product ion *m/z* 191.0>130.0 chromatogram of NCG in blank compound feed sample.

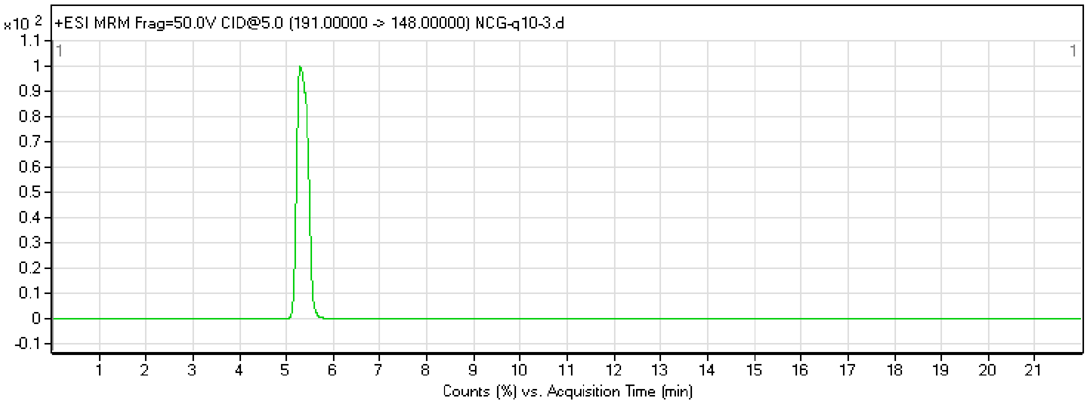

Figure S6. Product ion *m/z* 191.0>148.0 chromatogram of NCG in spiked compound feed.

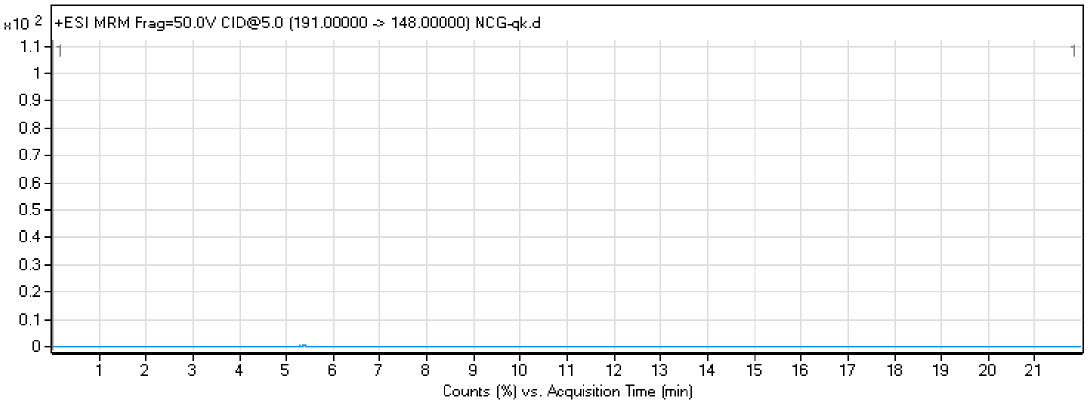

Figure S7. Product ion *m/z* 191.0>148.0 chromatogram of NCG in blank compound feed sample.

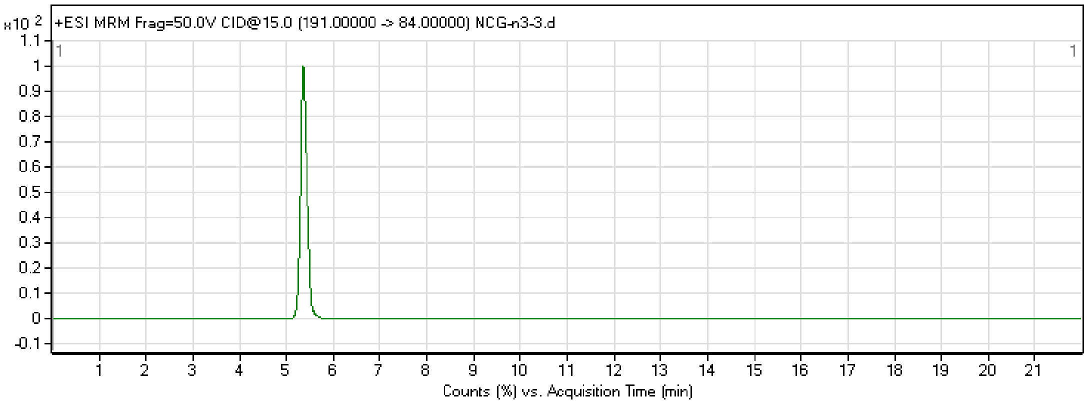

Figure S8. Product ion *m/z* 191.0>84.0 chromatogram of NCG in spiked concentrated feed.

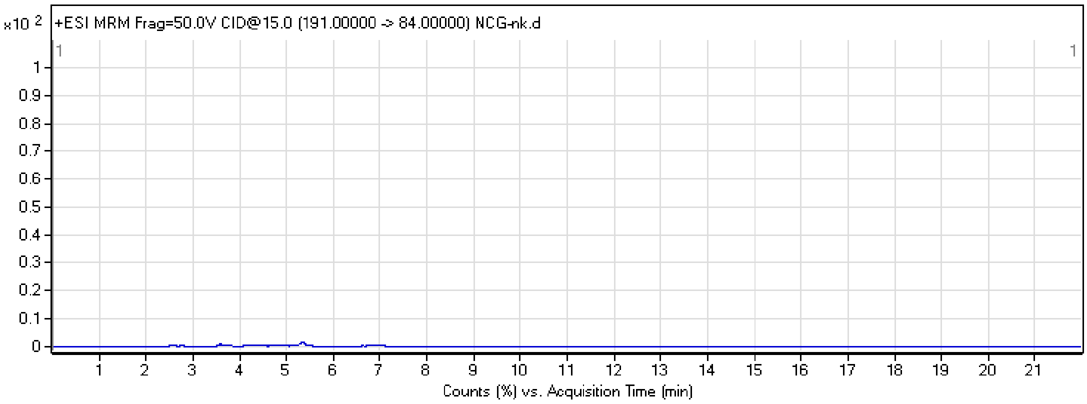

Figure S9. Product ion *m/z* 191.0>84.0 chromatogram of NCG in blank concentrated feed sample.

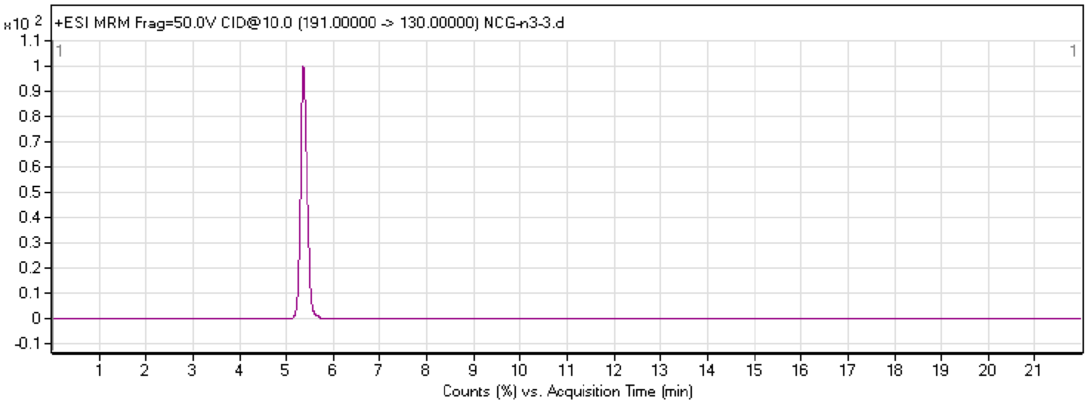

Figure S10. Product ion *m/z* 191.0>130.0 chromatogram of NCG in spiked concentrated feed.

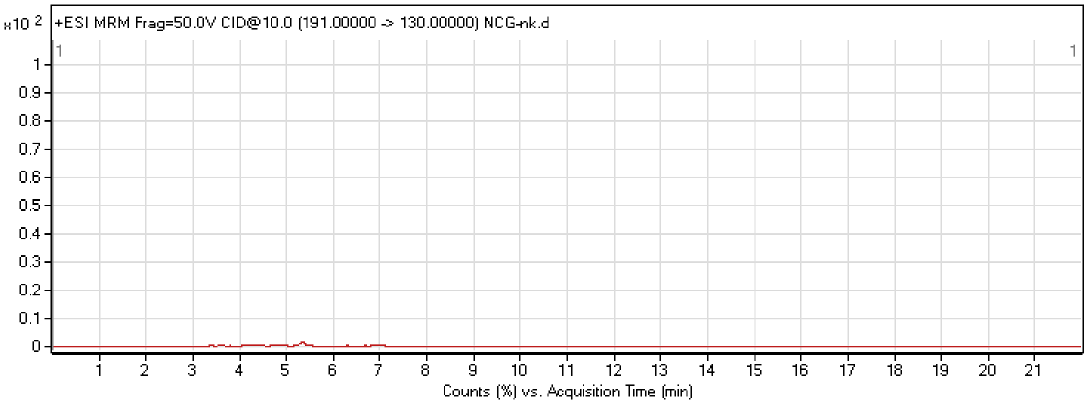

Figure S11. Product ion *m/z* 191.0>130.0 chromatogram of NCG in blank concentrated feed sample.

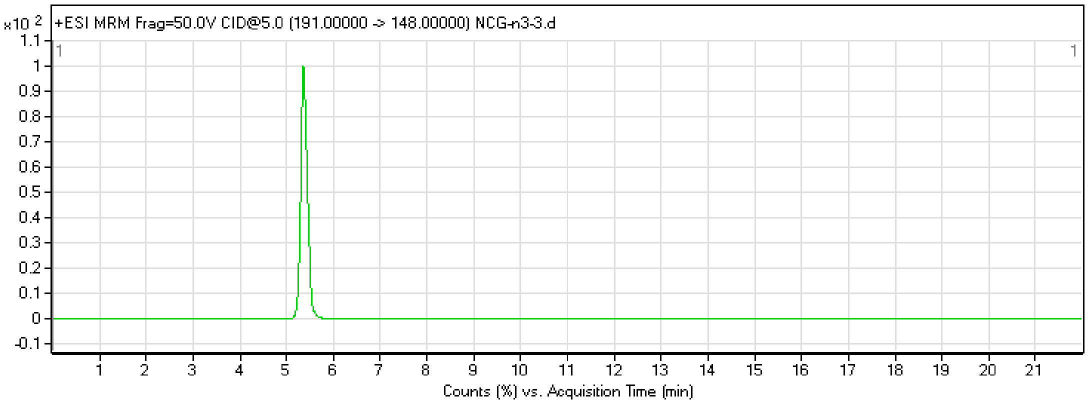

Figure S12. Product ion *m/z* 191.0>148.0 chromatogram of NCG in spiked concentrated feed.

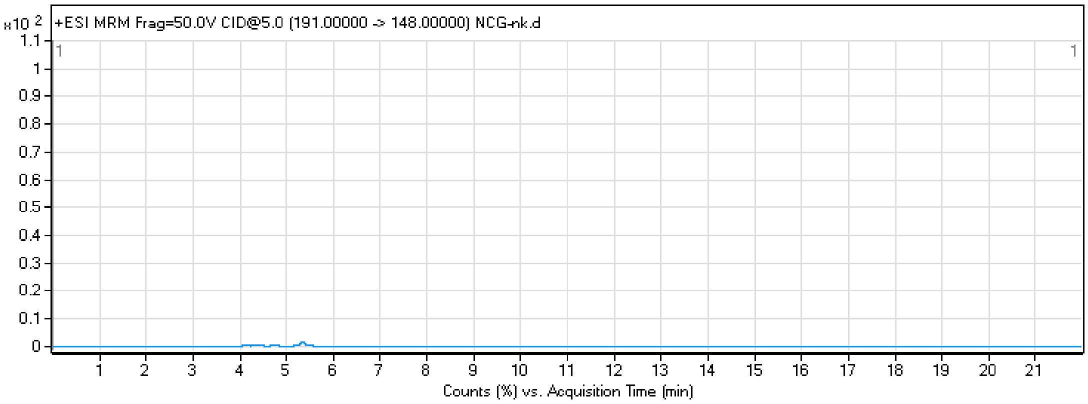

Figure S13. Product ion *m/z* 191.0>148.0 chromatogram of NCG in blank concentrated feed sample.

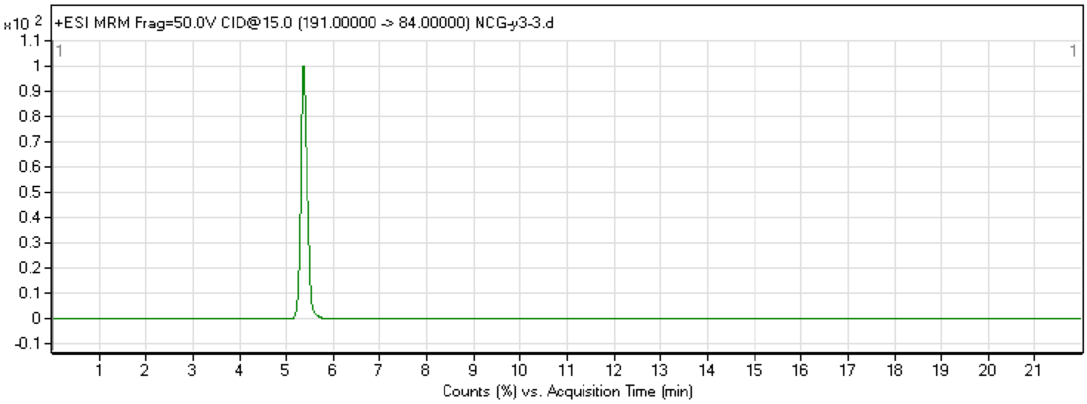

Figure S14. Product ion *m/z* 191.0>84.0 chromatogram of NCG in spiked premix.

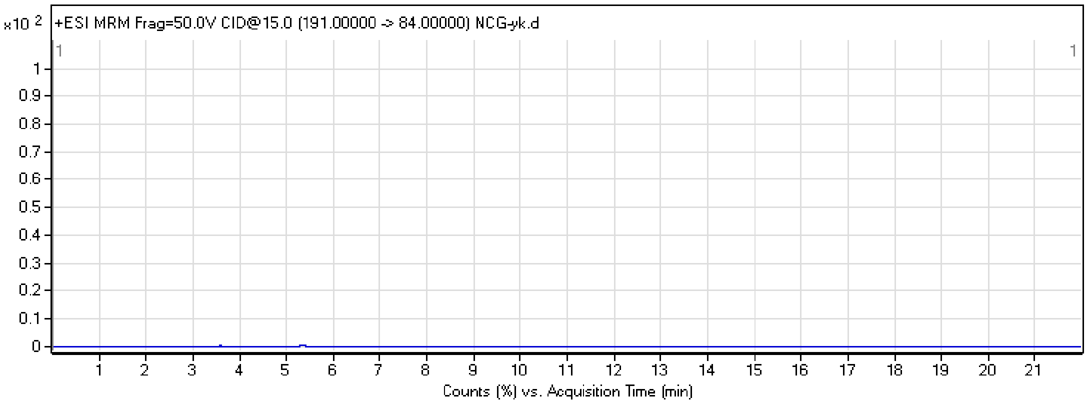

Figure S15. Product ion *m/z* 191.0>84.0 chromatogram of NCG in blank premix sample.

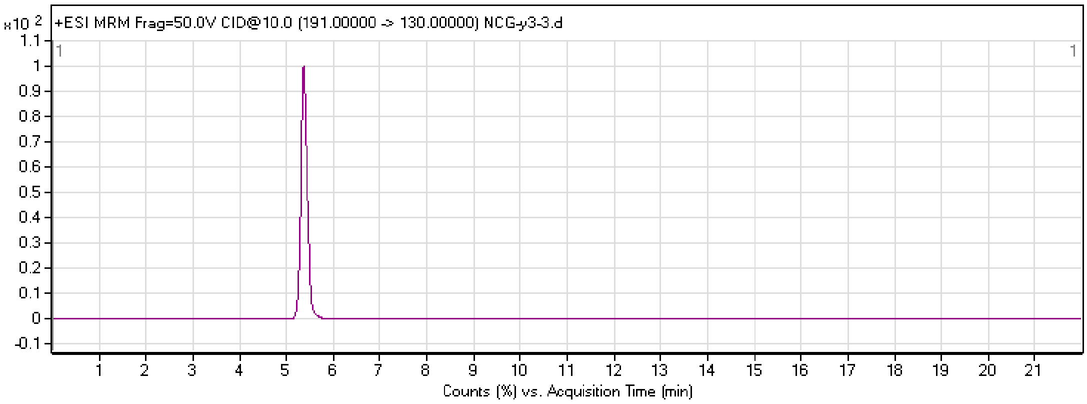

Figure S16. Product ion *m/z* 191.0>130.0 chromatogram of NCG in spiked premix.

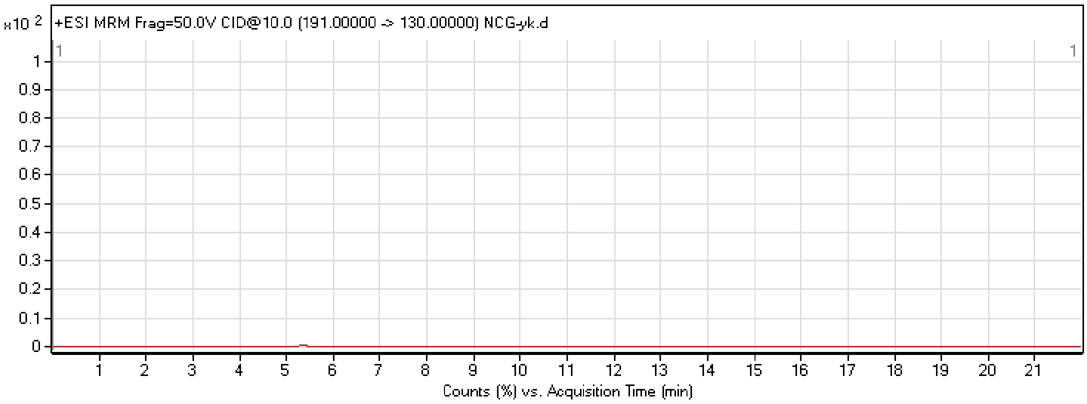

Figure S17. Product ion *m/z* 191.0>130.0 chromatogram of NCG in blank premix sample.

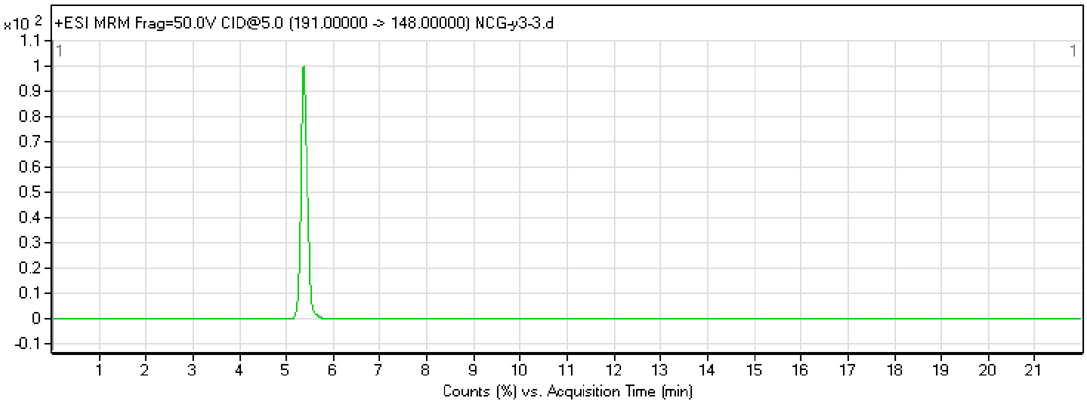

Figure S18. Product ion *m/z* 191.0>148.0 chromatogram of NCG in spiked premix.

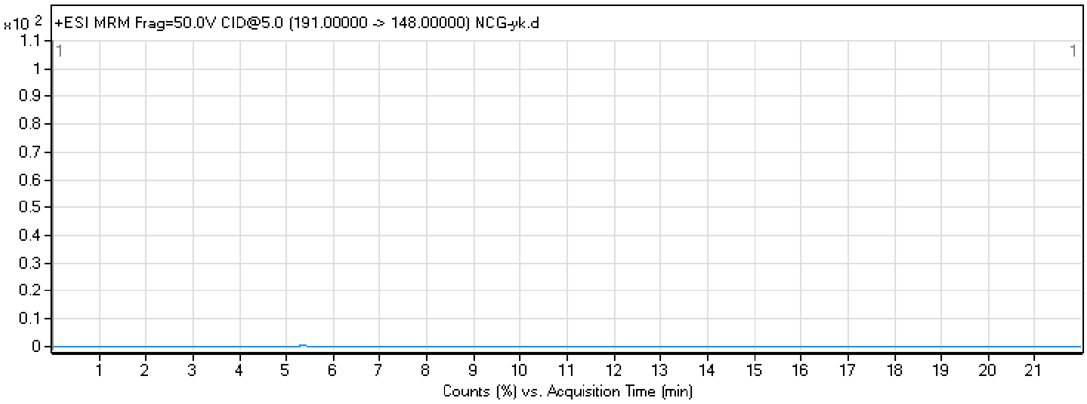

Figure S19. Product ion *m/z* 191.0>148.0 chromatogram of NCG in blank premix sample.

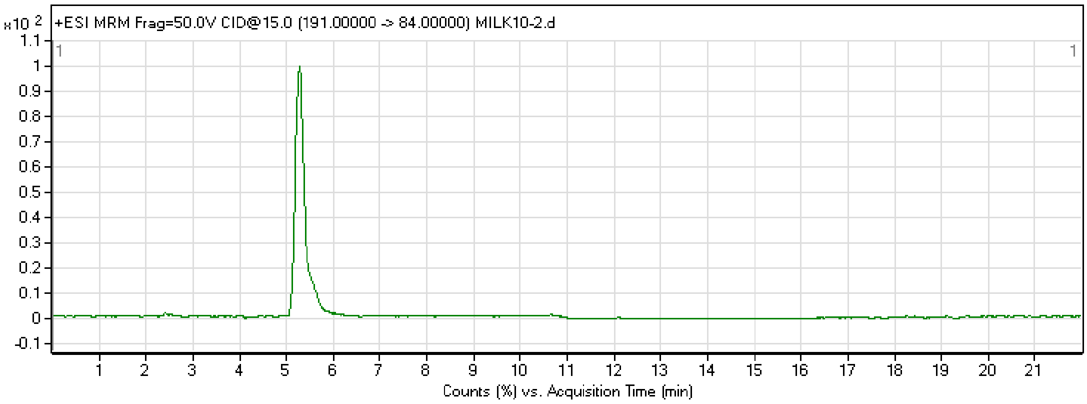

Figure S20. Product ion *m/z* 191.0>84.0 chromatogram of NCG in spiked milk.

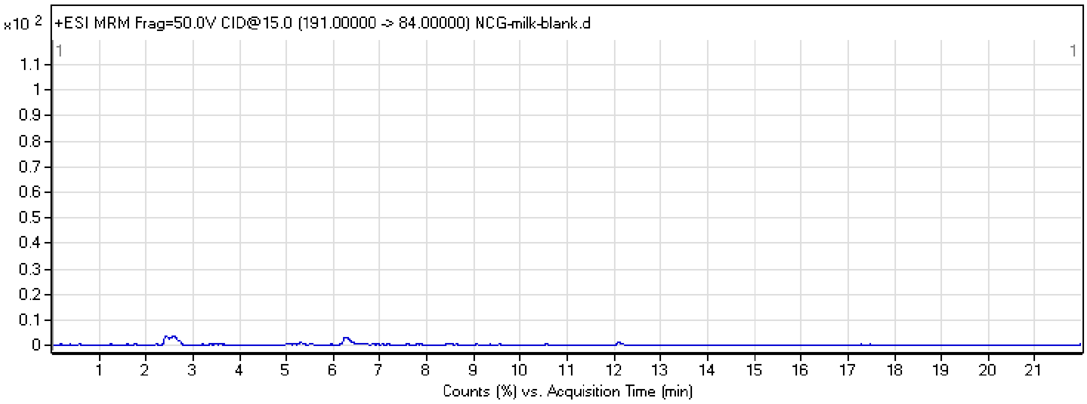

Figure S21. Product ion *m/z* 191.0>84.0 chromatogram of NCG in blank milk sample.

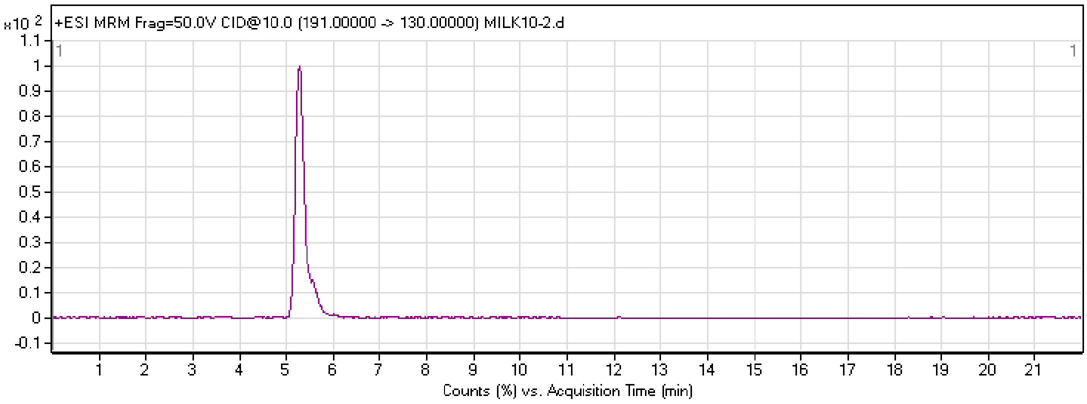

Figure S22. Product ion *m/z* 191.0>130.0 chromatogram of NCG in spiked milk.

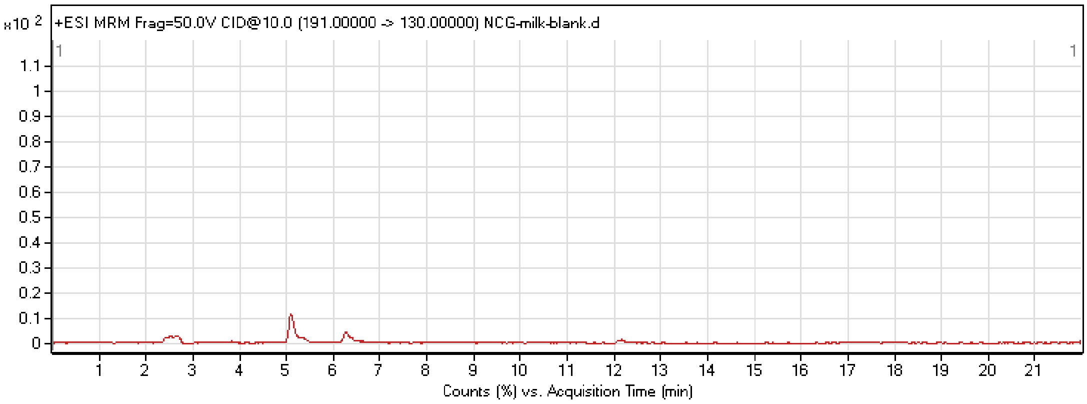

Figure S23. Product ion *m/z* 191.0>130.0 chromatogram of NCG in blank milk sample.

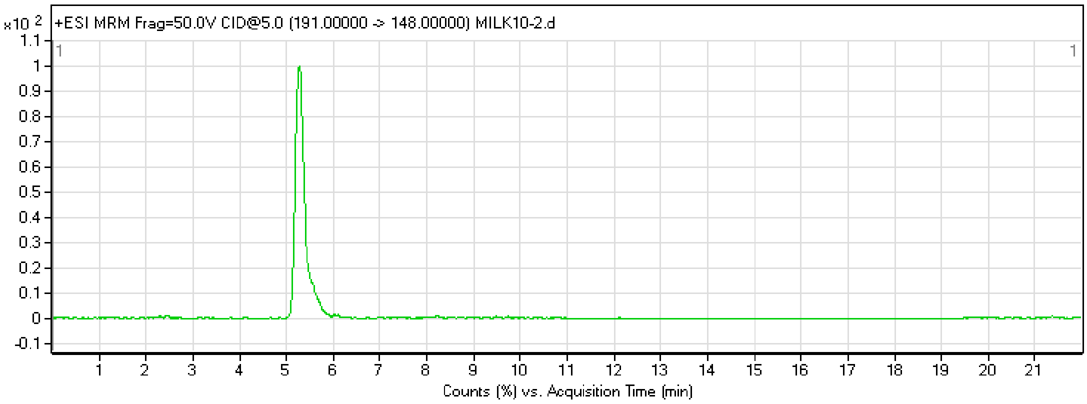

Figure S24. Product ion *m/z* 191.0>148.0 chromatogram of NCG in spiked milk.

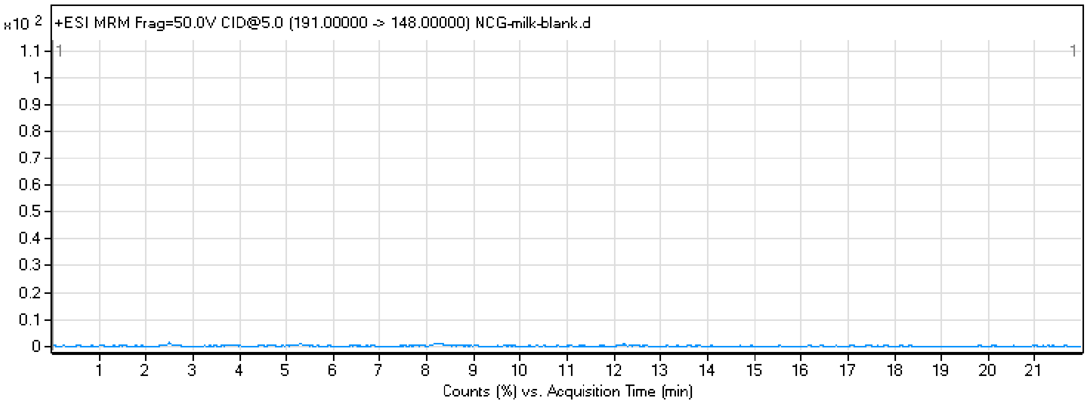

Figure S25. Product ion *m/z* 191.0>148.0 chromatogram of NCG in blank milk sample.

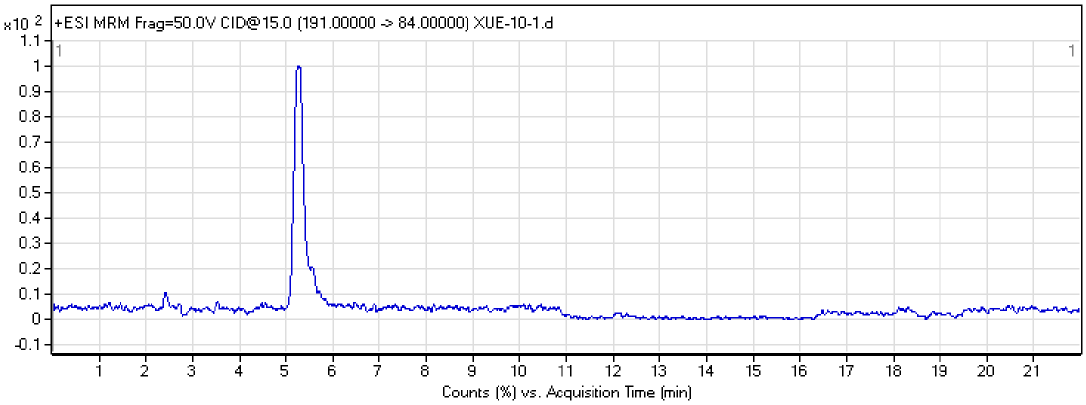

Figure S26. Product ion *m/z* 191.0>84.0 chromatogram of NCG in spiked serum.

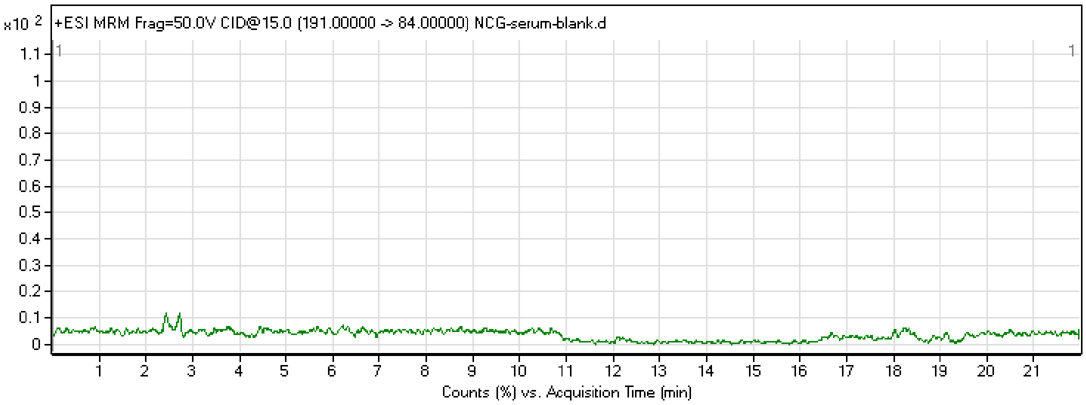

Figure S27. Product ion *m/z* 191.0>84.0 chromatogram of NCG in blank serum sample.

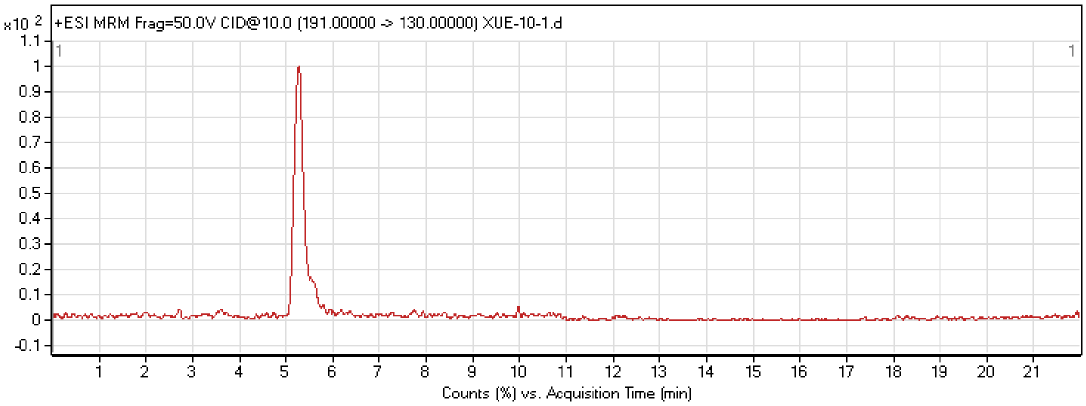

Figure S28. Product ion *m/z* 191.0>130.0 chromatogram of NCG in spiked serum.

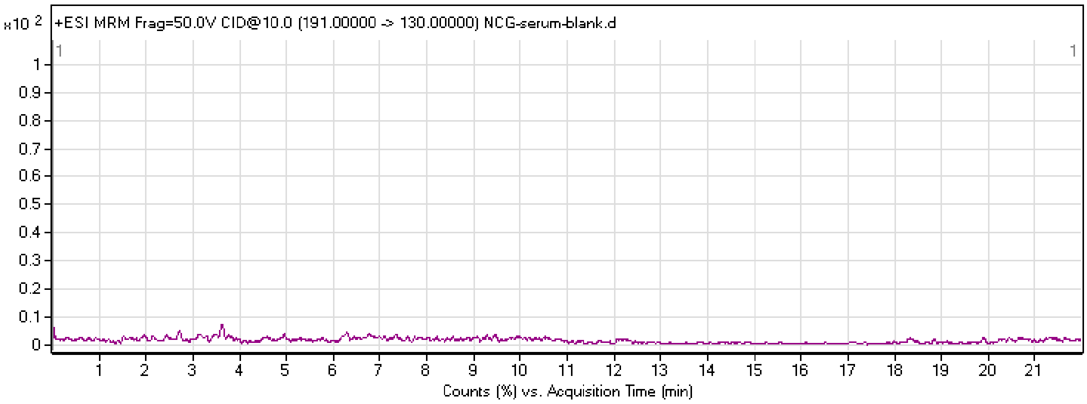

Figure S29. Product ion *m/z* 191.0>130.0 chromatogram of NCG in blank serum sample.

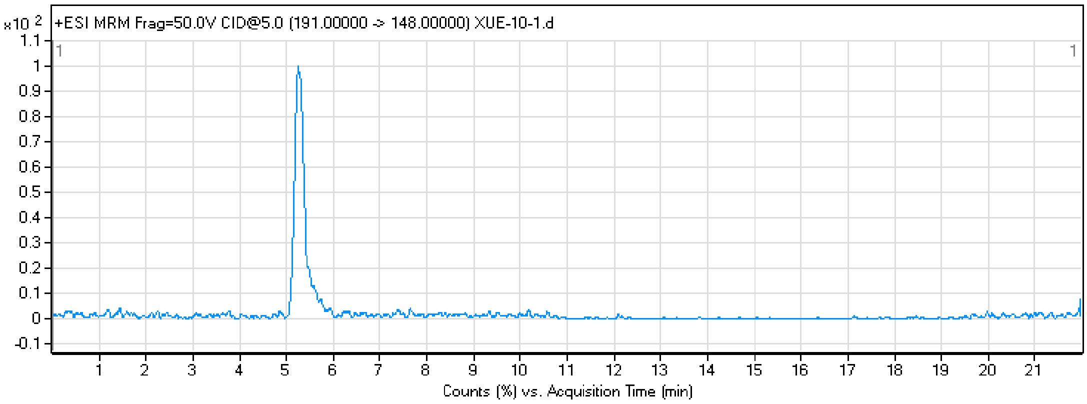

Figure S30. Product ion *m/z* 191.0>148.0 chromatogram of NCG in spiked serum.

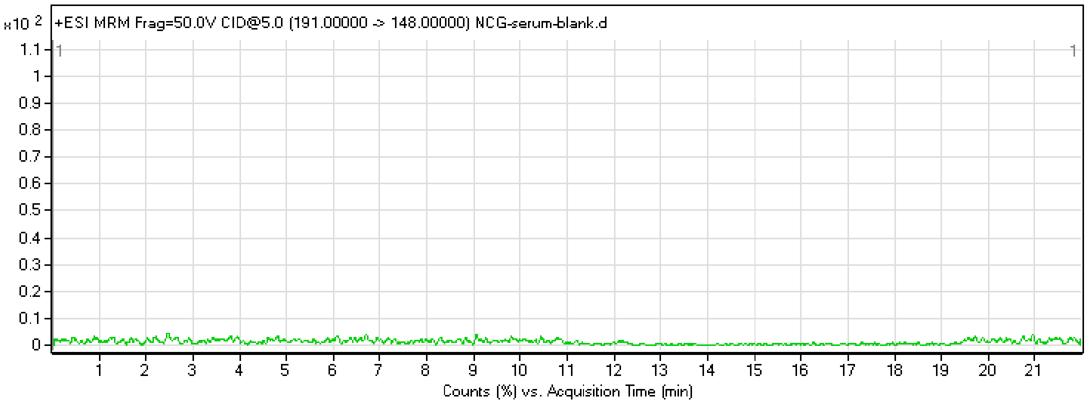

Figure S31. Product ion *m/z* 191.0>148.0 chromatogram of NCG in blank serum sample.

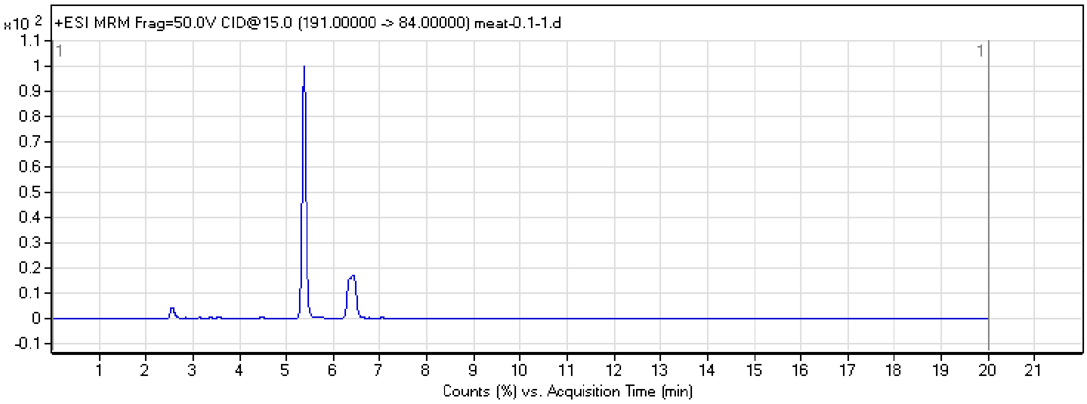

Figure S32. Product ion *m/z* 191.0>84.0 chromatogram of NCG in spiked meat.

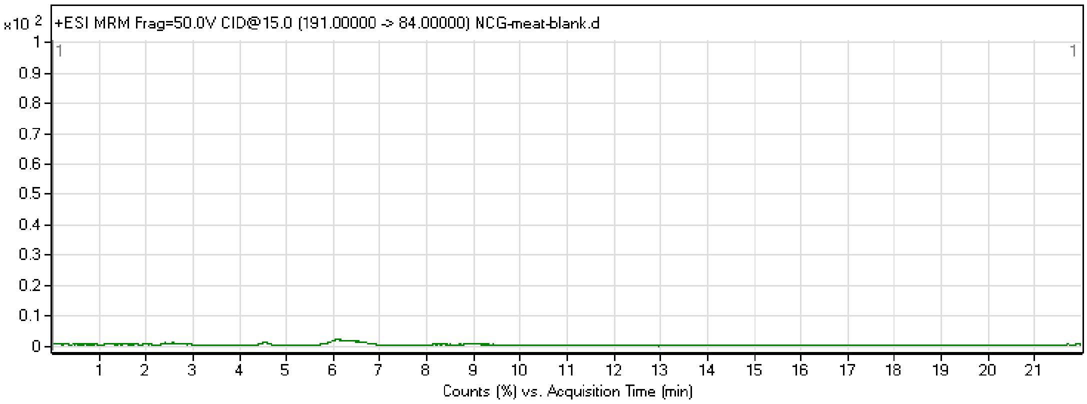

Figure S33. Product ion *m/z* 191.0>84.0 chromatogram of NCG in blank meat sample.

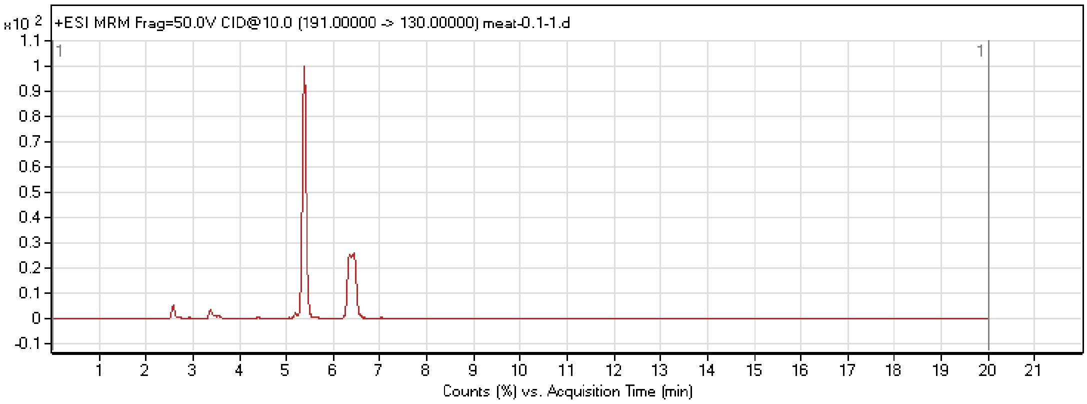

Figure S34. Product ion *m/z* 191.0>130.0 chromatogram of NCG in spiked meat.

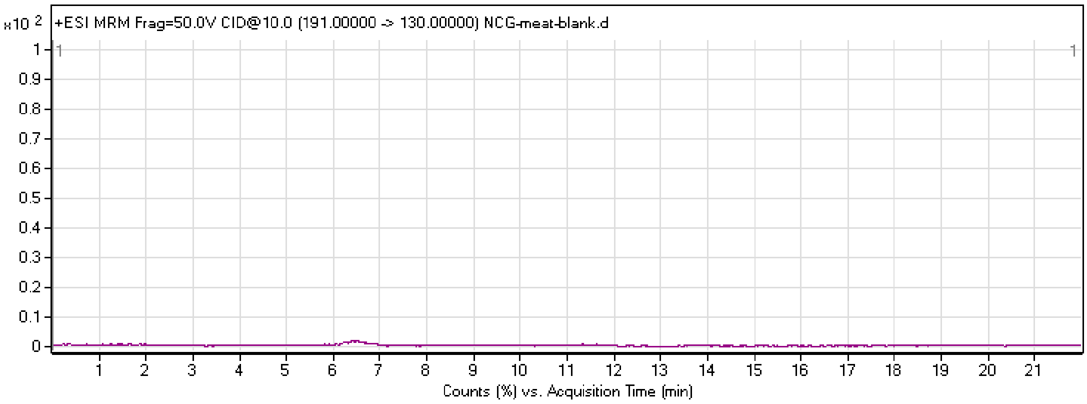

Figure S35. Product ion *m/z* 191.0>130.0 chromatogram of NCG in blank meat sample.

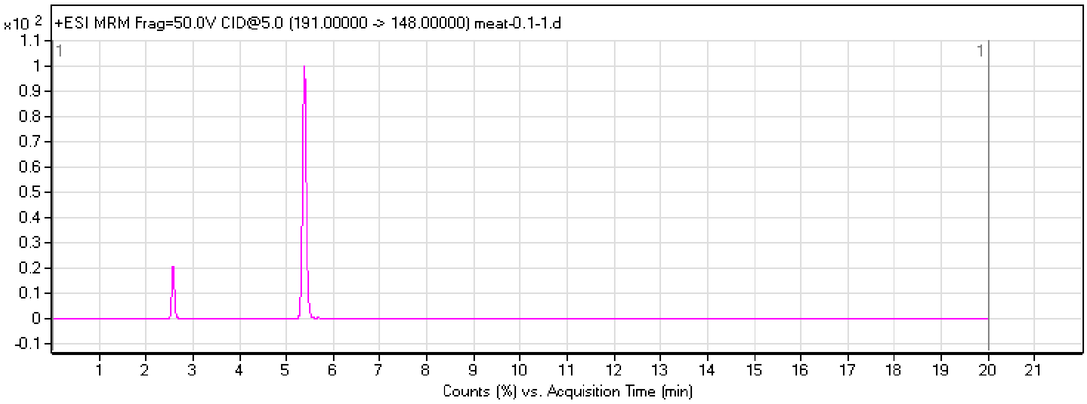

Figure S36. Product ion *m/z* 191.0>148.0 chromatogram of NCG in spiked meat.

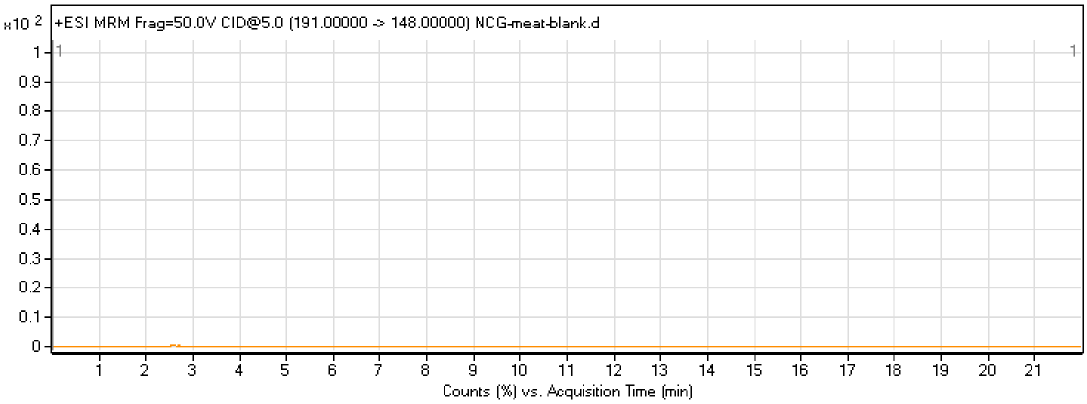

Figure S37. Product ion *m/z* 191.0>148.0 chromatogram of NCG in blank meat sample.

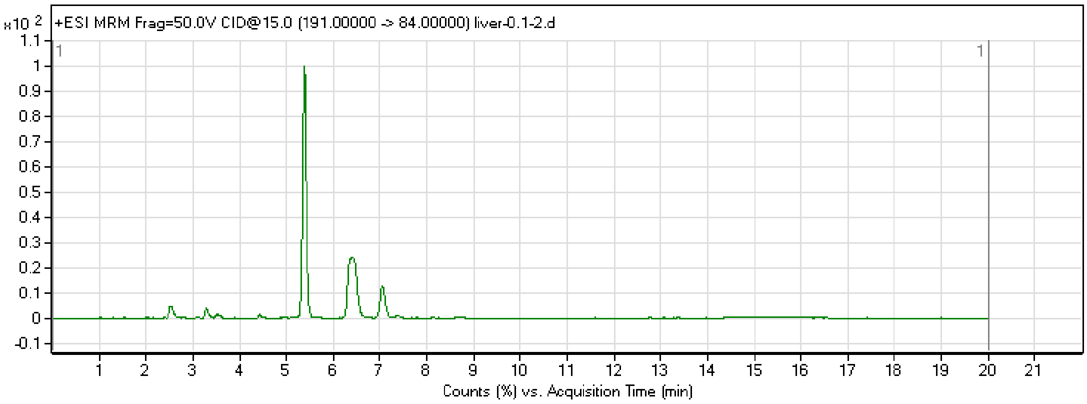

Figure S38. Product ion *m/z* 191.0>84.0 chromatogram of NCG in spiked liver.

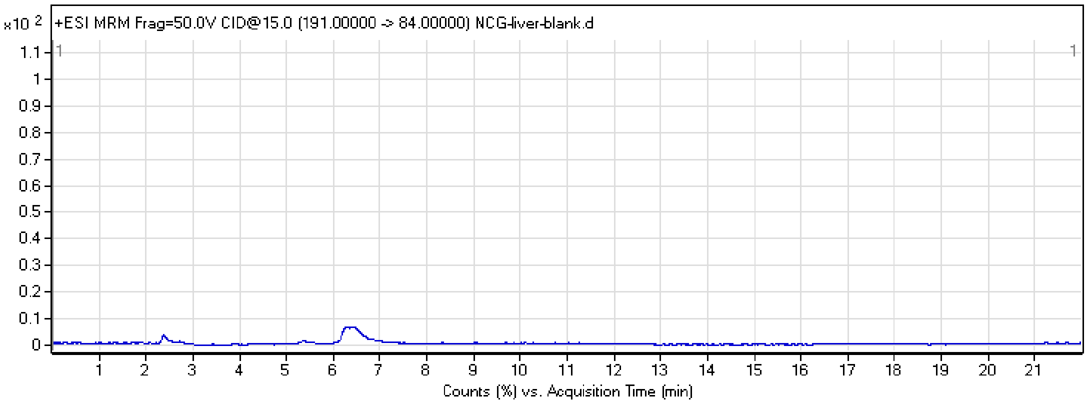

Figure S39. Product ion *m/z* 191.0>84.0 chromatogram of NCG in blank liver sample.

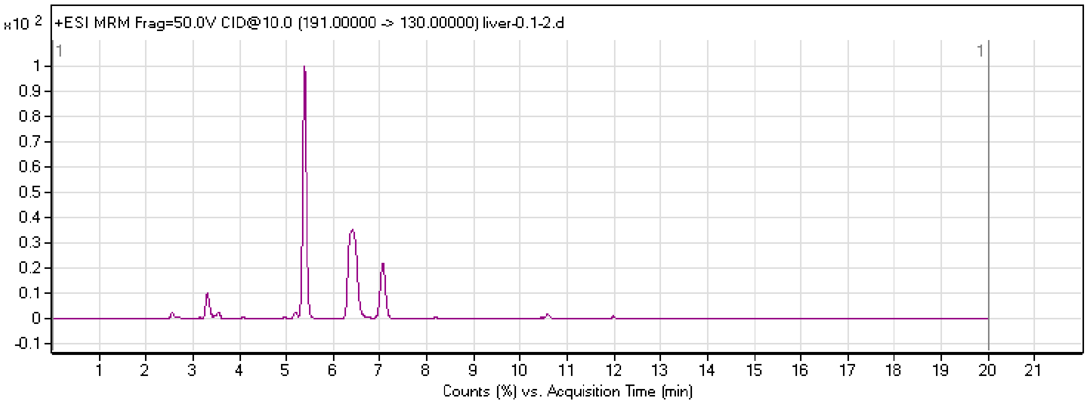

Figure S40. Product ion *m/z* 191.0>130.0 chromatogram of NCG in spiked liver.

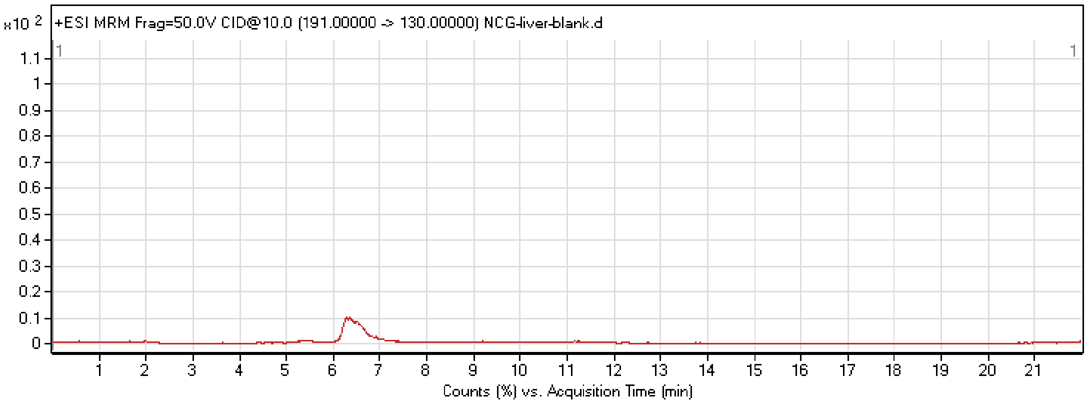

Figure S41. Product ion *m/z* 191.0>130.0 chromatogram of NCG in blank liver sample.

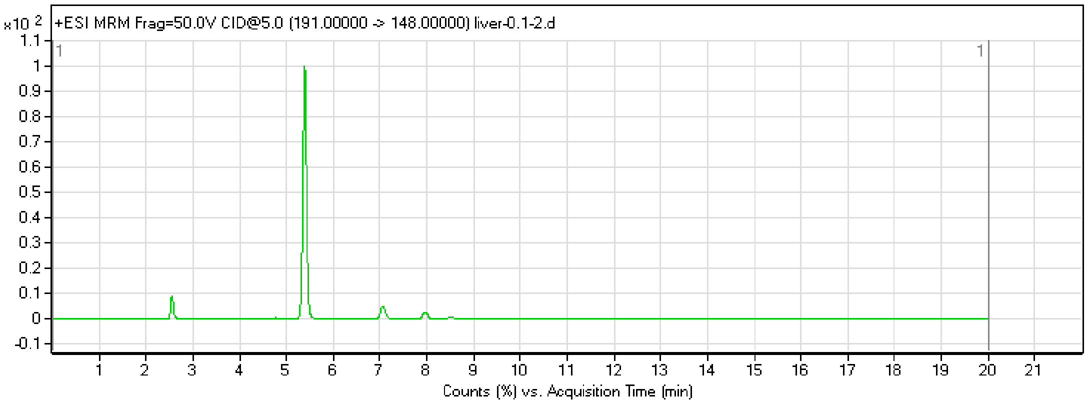

Figure S42. Product ion *m/z* 191.0>148.0 chromatogram of NCG in spiked liver.

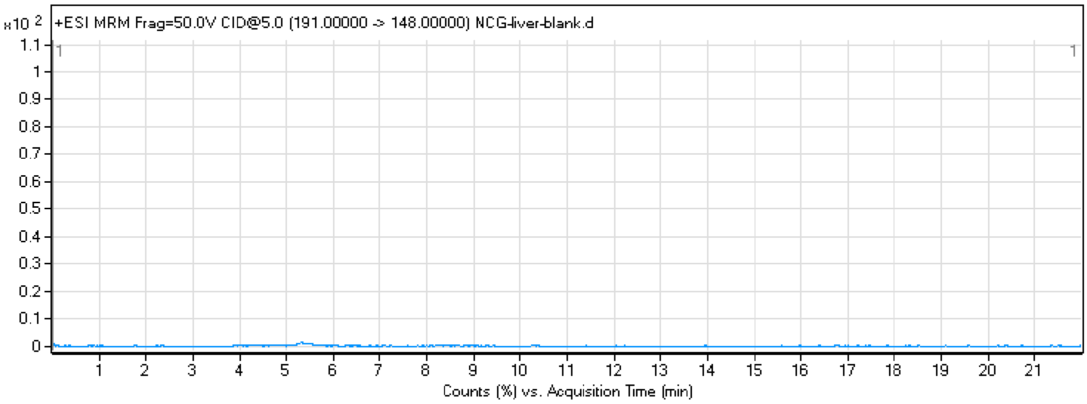

Figure S43. Product ion *m/z* 191.0>148.0 chromatogram of NCG in blank liver sample.

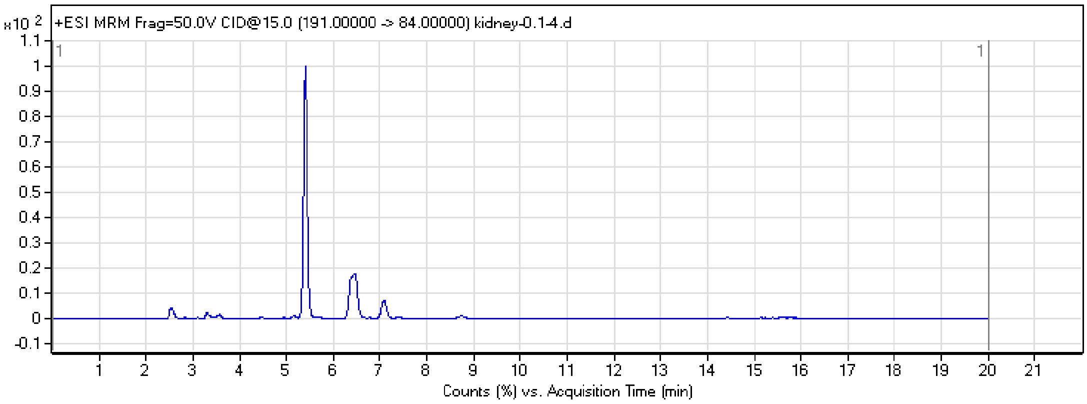

Figure S44. Product ion *m/z* 191.0>84.0 chromatogram of NCG in spiked kidney.

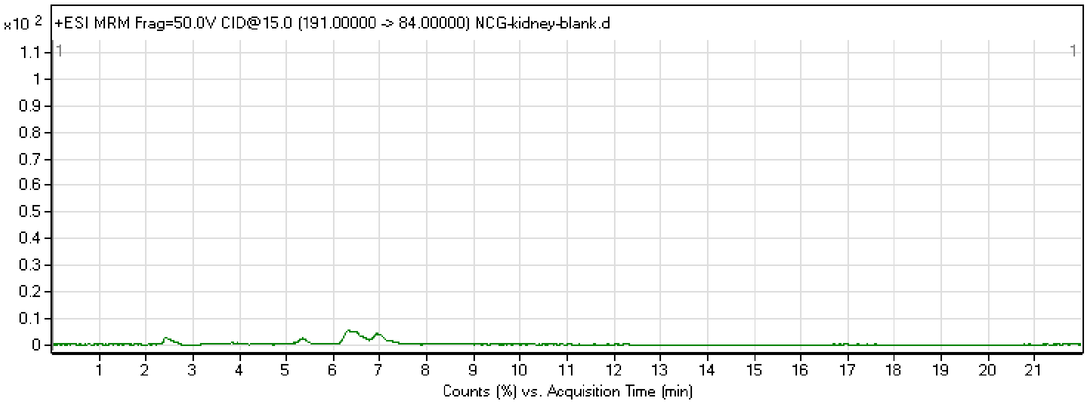

Figure S45. Product ion *m/z* 191.0>84.0 chromatogram of NCG in blank kidney sample.

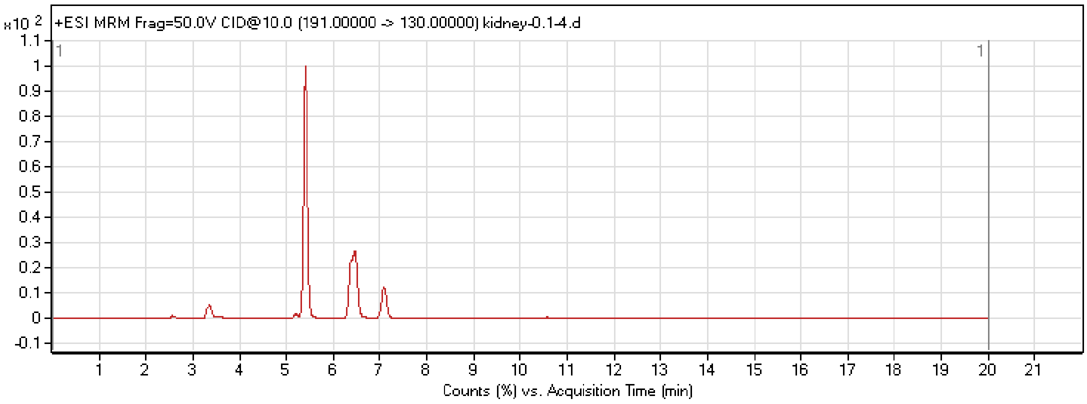

Figure S46. Product ion *m/z* 191.0>130.0 chromatogram of NCG in spiked kidney.

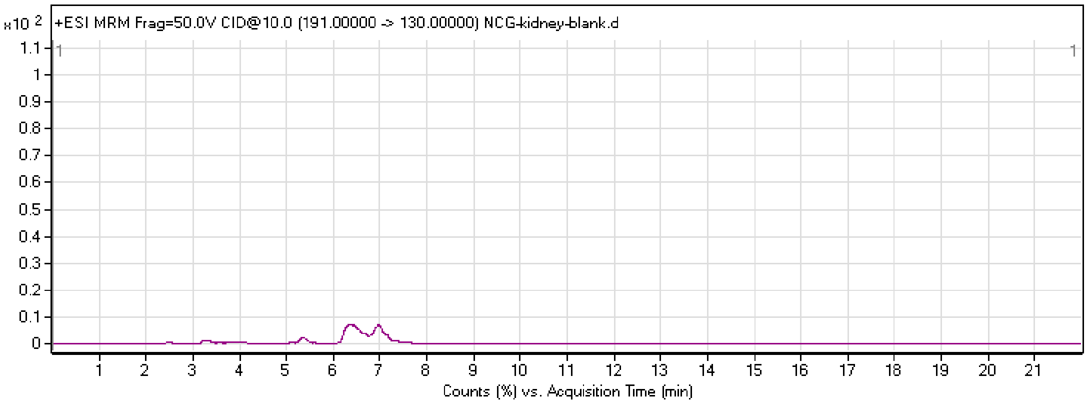

Figure S47. Product ion *m/z* 191.0>130.0 chromatogram of NCG in blank kidney sample.

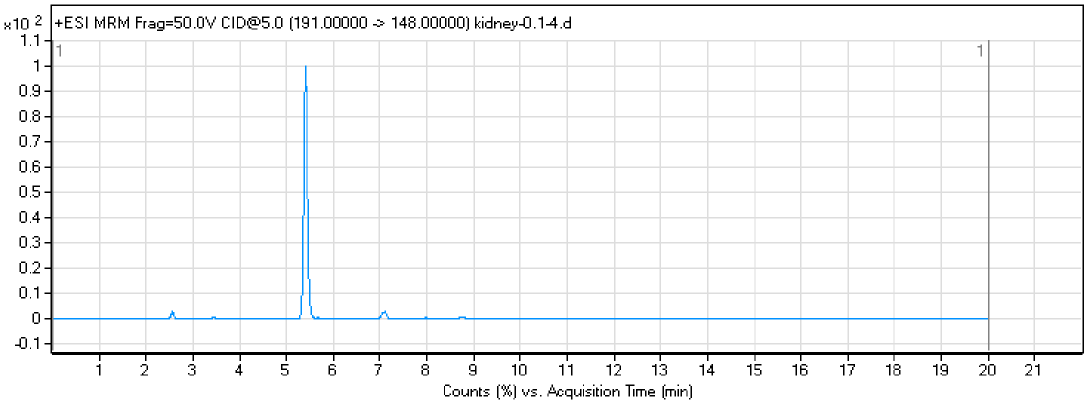

Figure S48. Product ion *m/z* 191.0>148.0 chromatogram of NCG in spiked kidney.

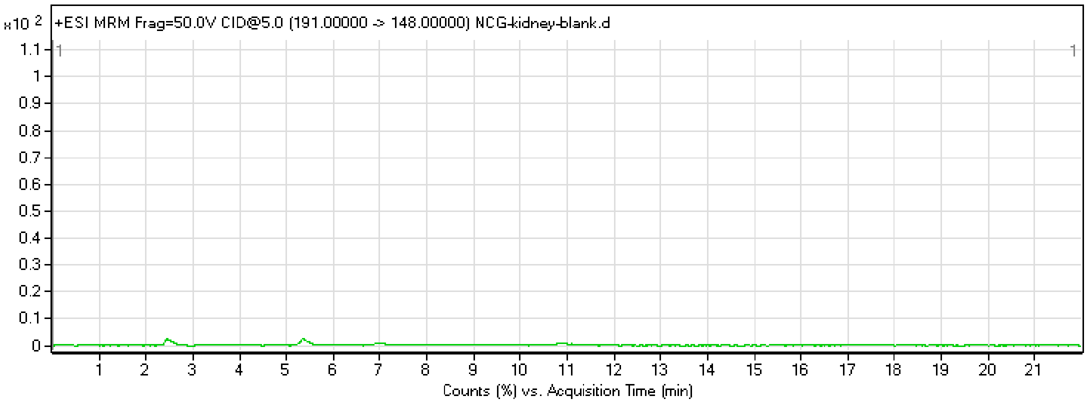

Figure S49. Product ion *m/z* 191.0>148.0 chromatogram of NCG in blank kidney sample.
